# Cigarette smoking and SARS-CoV-2 infection: multivariable regression and Mendelian randomization analyses in the Norwegian Mother, Father and Child Cohort Study

**DOI:** 10.1186/s12879-026-12750-8

**Published:** 2026-02-04

**Authors:** Ida Henriette Caspersen, Álvaro Hernáez, Sebastián Peña, Ahmed Nabil Shaaban, Maria Christine Magnus, Sakari Karvonen, Maria Rosaria Galanti, Per Magnus

**Affiliations:** 1 Centre for Fertility and Health, Norwegian Institute of Public Health, Skøyen, Postbox 222, Oslo, N-0213 Norway; 2 Blanquerna School of Health Sciences, University Ramon Llull, Barcelona, 08025 Spain; 3 CIBER Enfermedades Cardiovasculares (CIBERCV), Instituto de Salud Carlos III, Madrid, 28029 Spain; 4 Department of Public Health and Welfare, Finnish Institute for Health and Welfare, Postbox 30, Helsinki, 00271 Finland; 5 Department of Global Public Health, Karolinska Institutet, Stockholm, SE-171 77 Sweden; 6 Centre for Epidemiology and Community Medicine, Stockholm Region, Solnavägen 1E (Torsplan), Stockholm, 113 65 Sweden

**Keywords:** The Norwegian Mother and Child Cohort Study, MoBa, COVID-19, SARS-CoV-2

## Abstract

**Introduction:**

Evidence of whether smoking affects the risk for SARS-CoV-2 infection is mixed. We aimed to clarify the inconsistencies in previous findings and whether the association is potentially causal using different Mendelian randomization (MR) methods.

**Methods:**

We examined associations between smoking traits (initiation, cessation, and intensity) and SARS-CoV-2 infection in multivariable logistic regression, and one-sample and two-sample MR analyses. The study included *n* = 47,506 female and *n* = 28,229 male study subjects from the Norwegian Mother, Father, and Child Cohort Study (MoBa) with questionnaire data and genotype information. SARS-CoV-2 infection status was obtained by data linkage to the national health registry MSIS.

**Results:**

We found no clear evidence of an association between smoking initiation and SARS-CoV-2 infection (multivariable regression: OR 1.08, 95% CI 0.96 to 1.20, one-sample multivariable MR analysis: OR 1.02, 95% CI 0.96 to 1.09, two-sample MR: OR 1.10 (95% CI 1.06 to 1.13). Also, we found no clear evidence of an association with smoking intensity (multivariable regression: OR 0.78, 95% CI 0.62 to 0.96, one-sample multivariable MR: OR 1.04, 95% CI 0.76 to 1.42, two-sample MR: OR 1.01, 95% CI 0.95 to 1.09, per 1 SD increase in number of cigarettes per day). Nor was there any association with smoking cessation. These findings did not change after accounting for educational level, BMI or risk-taking behavior in multivariable MR analyses.

**Conclusions:**

We did not find robust evidence of causal associations between smoking and SARS-CoV-2 infection. Our investigation of potential violations to MR assumptions highlights the limitations of this approach to examine infection risk associated with smoking.

**Supplementary Information:**

The online version contains supplementary material available at 10.1186/s12879-026-12750-8.

## Introduction

There is robust evidence that smokers who were infected with SARS-CoV-2 during the first years of the pandemic had increased risk of severe disease progression and death from COVID-19 [1, 2], as well as increased risk of long-lasting sequelae [3]. However, conventional observational studies showed that current smokers have lower risk of testing positive for SARS-CoV-2 infection than non-smokers [4]. In the Nordic countries, we found similar inverse associations in studies based on Finnish, Swedish and Norwegian general population samples [5–7].

To clarify whether associations between smoking and COVID-19 outcomes are causal, a combination of methods with distinct strengths and biases is useful [8]. Mendelian randomization (MR) studies, which use genetic variants associated with smoking traits (e.g. ever smoking) to evaluate the unconfounded impact of this exposure on a specific outcome (e.g., SARS-CoV-2 infection) [9] under the assumption that genetic variants are randomly distributed in unselected populations. Several MR analyses have been conducted to investigate associations between smoking and various COVID-19 outcomes, some also including confirmed SARS-CoV-2 infection as an outcome [10–15]. Interestingly, the MR studies have suggested a positive association between genetically predicted smoking traits and risk of confirmed SARS-CoV-2 infection [10, 11, 13]. For instance, an MR study based on the UK Biobank showed positive associations with SARS-CoV-2 infection for genetically predicted smoking initiation and smoking heaviness among ever smokers. However, in standard multivariable analyses in the same study, a positive association was seen only for former, but not current smokers [10].

The discrepancy between results from MR studies and standard observational studies has not been well explained. One explanation is residual confounding or collider bias in standard observational studies [16]. Another explanation is potential violation of underlying assumptions of the MR analysis. MR results are less prone to confounding by socioeconomic and behavioral factors that generally bias conventional observational studies. However, MR studies may be biased by (1) weak instruments, (2) confounding by population stratification and (3) horizontal pleiotropy [17, 18]. To avoid bias by horizontal pleiotropy, it is essential that the genetic instrument is associated with the outcome only through the exposure in question [17]. Specifically, it has been suggested that risk-taking behavior, which may share genetic risk factors with smoking [19], may represent a violation of the pleiotropy assumption in previous MR studies [10].

Understanding the reasons behind conflicting evidence on the health effects of smoking is essential for effectively communicating its risks, including during future pandemics. Our objective was therefore to investigate the association between smoking traits and risk of SARS-CoV-2 infection using standard multivariable regression and MR analyses, taking potential sources of horizontal pleiotropy (such as risk-taking behavior) into account in the MR analysis.

## Methods

### Study participants

We used data from participants in the Norwegian Mother, Father, and Child Cohort Study (MoBa), a prospective, population-based, pregnancy cohort conducted by the Norwegian Institute of Public Health [20]. Pregnant mothers and their partners were recruited from all over Norway between 1999 and 2008. The cohort comprises around 95,000 mothers, 75,000 fathers, and 114,000 children. Since March 2020, all adult participants in MoBa have been regularly invited to answer electronic questionnaires regarding illness, testing for SARS-CoV-2, lifestyle during the pandemic and more. The response rates to questionnaires distributed since March 2020 have been 50–80%. We used version 12 of the quality-assured MoBa data files released on May 11, 2022.

For this study, we used a subset of MoBa mothers and fathers with information on genotype and tobacco use. Genotype data was procured from blood samples taken during pregnancy [21]. In MoBa, samples from mothers, fathers and children have been genotyped across 24 genotyping batches, each with distinct selection criteria, genotyping platforms, and genotyping centers [22]. Genotype calling, quality control, phasing, and imputation were carried out using the MoBaPsychGen pipeline, developed by Corfield et al. [22]. Our study adheres to the Strengthening the Reporting of Observational Studies in Epidemiology (STROBE) guidelines for MR and cohort studies [23, 24].

### Smoking variables

MoBa parents answered questions related to their smoking behavior at recruitment (1999–2008), during follow-up (our study included data on smoking up to 8 years after recruitment, and smoking status in 2015, for men only), and after the pandemic outbreak, in June 2020 and January 2021. Participants were asked at several timepoints whether they had ever smoked or were currently smoking. We defined “ever smoking” as having at least one report of prior or current smoking in any MoBa questionnaire. We defined current smoking based on the questionnaire in the beginning of the pandemic (June 2020), or, if missing, at the next available time point, in January 2021 (9% of the sample). We defined three exposure variables: (1) smoking initiation, defined as having ever smoked (yes/no, applicable to the entire sample); (2) smoking intensity, measured as the number of cigarettes currently smoked per day (continuous, applicable to current smokers; (3) smoking cessation, defined as having ever smoked, but not currently smoking in June 2020 or January 2021 (yes/no, applicable to former or current regular smokers).

### Genetic instruments for smoking traits

Genetic instruments were derived from the genome-wide association study (GWAS) on smoking-related traits by Liu and colleagues [25]. This study encompassed over 1.2 million participants (with no overlap with MoBa) and identified 378 distinct single nucleotide polymorphisms (SNPs) associated with having ever been a regular smoker (smoking initiation), 55 related to the number of cigarettes smoked per day (smoking intensity), and 24 linked to quitting smoking [25]. Independent SNPs were determined based on linkage disequilibrium blocks throughout the genome, exhibited a minor allele frequency of at least 0.1%, and showed association with their corresponding phenotypes as per the standard genome-wide significance threshold (*p*-value < 5 × 10^− 8^) [25]. In our database, 351 (93%) of the SNPs from the Liu M et al. GWAS were accessible for smoking initiation, 53 (96%) for smoking intensity, and 22 (92%) for smoking cessation.

In the one-sample MR analyses, we produced weighted genetic risk scores (GRS, i.e., polygenic scores) by multiplying the count of risk alleles with the effect estimate for each variant, then dividing the result by the total SNP count [26]. In the two-sample MR, SNPs were used individually as genetic instruments. Details regarding which SNPs were involved in both one-sample and two-sample MR analyses can be found in Supplementary Tables S1-S3.

### SARS-CoV-2 infection

The outcome, having acquired a SARS-CoV-2 infection, was measured as a laboratory-confirmed positive PCR test for SARS-CoV-2, registered in the period March 6th, 2020 to May 25th, 2021. This time window covers the pre- and early vaccination period of the pandemic in Norway and resembles the period covered in a previous study on current tobacco use and SARS-CoV-2 infection in the same population [6]. This time window thus allows investigation of associations between smoking and SARS-CoV-2 infection risk without the presence of intermediate effects of vaccine hesitancy and/or effectiveness. Information on all positive PCR tests were obtained by data linkage to The Norwegian Surveillance System for Communicable Diseases (MSIS).

### Other variables

Information about the participant’s gender was based on their cohort member role as mother or father. Data on participants’ age at follow-up in 2020 (continuous) was calculated from birth year in The Medical Birth Registry. Region of residence (Oslo and metropolitan region-Viken, Southern Norway, Western Norway, Eastern Norway, Northern Norway) and number of cohabitants (discrete) were assessed by questionnaires in 2020. Education level (< High school; High school; College, ≤ 4 years; College, > 4 years) was assessed by questionnaire in 2021, and missing registrations were replaced by education level registered at recruitment in MoBa. The number of cohabitants were included as a proxy of social contacts, under the assumption that this may have influenced both smoking behavior as well as the SARS-CoV-2 infection risk.

### Statistical analyses

#### Multivariable logistic regression

We evaluated the associations between smoking-related traits and SARS-CoV-2 infection using multivariable logistic regressions. Because all study participants were originally recruited as a couple and may still share households, we examined all associations both in the whole population and women and men separately. We tested for interactions between smoking traits and gender by including product terms in the regression models. For binary exposure variables such as smoking initiation (in the entire population) and smoking cessation (among ever smokers), we investigated the odds of SARS-CoV-2 between those exposed in comparison to those not exposed. For continuous variables (smoking intensity), we assessed the association between a 1 SD unit increase in the quantity of cigarettes smoked per day or in age at initiation with the odds of infection (among ever smokers). All models were run both without and after adjustment for pre-established covariates including age, region of residence, education level, and number of cohabitants. Covariates were selected a priori based on a hypothesized causal diagram. The proportion of subjects with missing information on covariates was low (< 3%), and models were run on complete cases.

#### One-sample MR

In the one-sample MR, we used a two-stage approach with the smoking trait genetic risk scores as instruments. First, we used logistic regression models to estimate the genetically predicted likelihood of smoking initiation (all participants) and smoking cessation (in ever smokers), and linear regression to compute the genetically predicted values of the number of cigarettes smoked per day. Subsequently, we assessed the association between these genetically predicted traits (per 1 SD increase) and SARS-CoV-2 infection using logistic regression models, adjusting for the first 20 ancestry-informative genetic principal components and genotype batch.

#### Two-sample MR

We used summary data from the largest GWAS of COVID-19 in populations of European ancestry from the COVID-19 Host Genetics Initiative website (https://www.covid19hg.org/results/r7/) (Release #7, *n* = 14,496,978) [27]. In the GWAS summary data for COVID-19, we searched for SNPs related to smoking initiation, intensity, and cessation, and extracted information regarding their association with COVID-19. After harmonizing both datasets and excluding palindromic SNPs with minor allele frequencies near 0.5, we used a random effects inverse variance weighted regression method as the primary two-sample MR analysis to estimate the association between the smoking-related trait of interest and SARS-CoV-2 infection [18].

#### Verification of MR assumptions

To ensure that MR results are valid, three key assumptions must be met: (1) the genetic instrument used is robustly related to the exposure of interest; (2) there is no confounding of the genetic instrument-outcome association due to population stratification; and (3) the genetic instrument only affects the outcome through the exposure of interest (no horizontal pleiotropy) [18].

To verify the first assumption, we evaluated the strength of the association between the genetic instruments and the exposures in the MoBa cohort. For binary exposures (smoking initiation and cessation), we used the area under the receiver operating characteristic curve, pseudo-R^2^ by the Nagelkerke method, and logistic regressions. For continuous exposures (smoking intensity), we used F-statistics, R^2^ and linear regressions. In addition, the risk of weak instrument bias was assumed to be low whenever one-sample and two-sample MR estimates agreed (one-sample MR tends to be biased away from the null/towards the standard observational estimate and two-sample MR tends to be biased towards the null) [28]. In addition, we estimated the association between the GRSs for smoking traits (smoking intensity and smoking cessation) and risk of SARS-CoV-2 infection in non-exposed participants (i.e., those reporting no current or prior smoking). As we would expect no association between the GRSs and the outcome in non-exposed, any evidence of one would indicate the presence of bias.

Regarding the second assumption, we reduced potential confounding due to population stratification by: (1) adjusting our one-sample MR analyses for the first 20 ancestry-informative principal components [29], and (2) using summary statistics from GWASs that adjusted their analyses for genetic principal components [18].

To assess the third assumption (to examine the presence of horizontal pleiotropy in our results), we first conducted various sensitivity analyses based on two-sample MR techniques (MR-Egger, weighted median, and weighted mode) [30]. Horizontal pleiotropy was identified through the following criteria: (1) evidence of a non-zero intercept in the MR-Egger method, as indicated by the p-value for the intercept term; (2) lack of concordance between MR estimates in inverse variance weighted and alternative methods; and (3) presence of SNP heterogeneity as indicated by Cochran’s Q and Rücker’s Q’ tests [30]. As several signs of horizontal pleiotropy were detected, we explored in depth the variables that could explain this pleiotropy. We evaluated the association of the GRSs for smoking-related traits with variables closely related to tobacco use for which we had information in our database, such as education years and BMI. There was evidence of one or more of the smoking trait GRS’ associating with both factors. We also assumed an association between the genetic variants linked to smoking and those linked to risk-taking behaviors, according to previous literature [31]. As GWAS summary statistics for the three potentially pleiotropic variables were available, we performed multivariable MR analyses [32]. In the multivariable analysis, standard MR assumptions hold, but are now conditional on all the included variables, and not on the smoking instrument alone. The estimate can therefore be interpreted as the direct effect of the exposure (adjusting for other exposures) on the outcome [32]. For education years, we used the GWAS by Lee JJ et al. (*n* = 1,271 independent SNPs, 1,176 of which were available in MoBa) [33]. For BMI, we used the GWAS by Yengo L et al. (*n* = 941 independent SNPs, 908 of which were available in MoBa) [34]. For risk-taking behaviors, we used the GWAS by Strawbridge RJ et al. (*n* = 2 independent SNPs, both available in MoBa) [31]. We generated GRSs for education years, BMI and the likelihood of presenting risk-taking behavior using the same method as used for the smoking traits [32] and then included the genetically predicted values for these covariates in the one-sample MR regression models. The genetic instruments for BMI and education attainment were robust. The GRS for education explained 4.1% of variance in education years in women and 3.9% of variance in men. For BMI, the GRS explained 5.7% of variance in BMI in women and 5.1% of variance in men. For risk-taking behavior, we did not have observational data and were therefore not able to evaluate the robustness of the genetic instrument.

Analyses were conducted in R Software v. 4.1.2. A pre-registered protocol is not available for this study. Code for one-sample and two-sample MR analysis is available in https://github.com/alvarohernaez/.

## Results

**Fig. 1:**
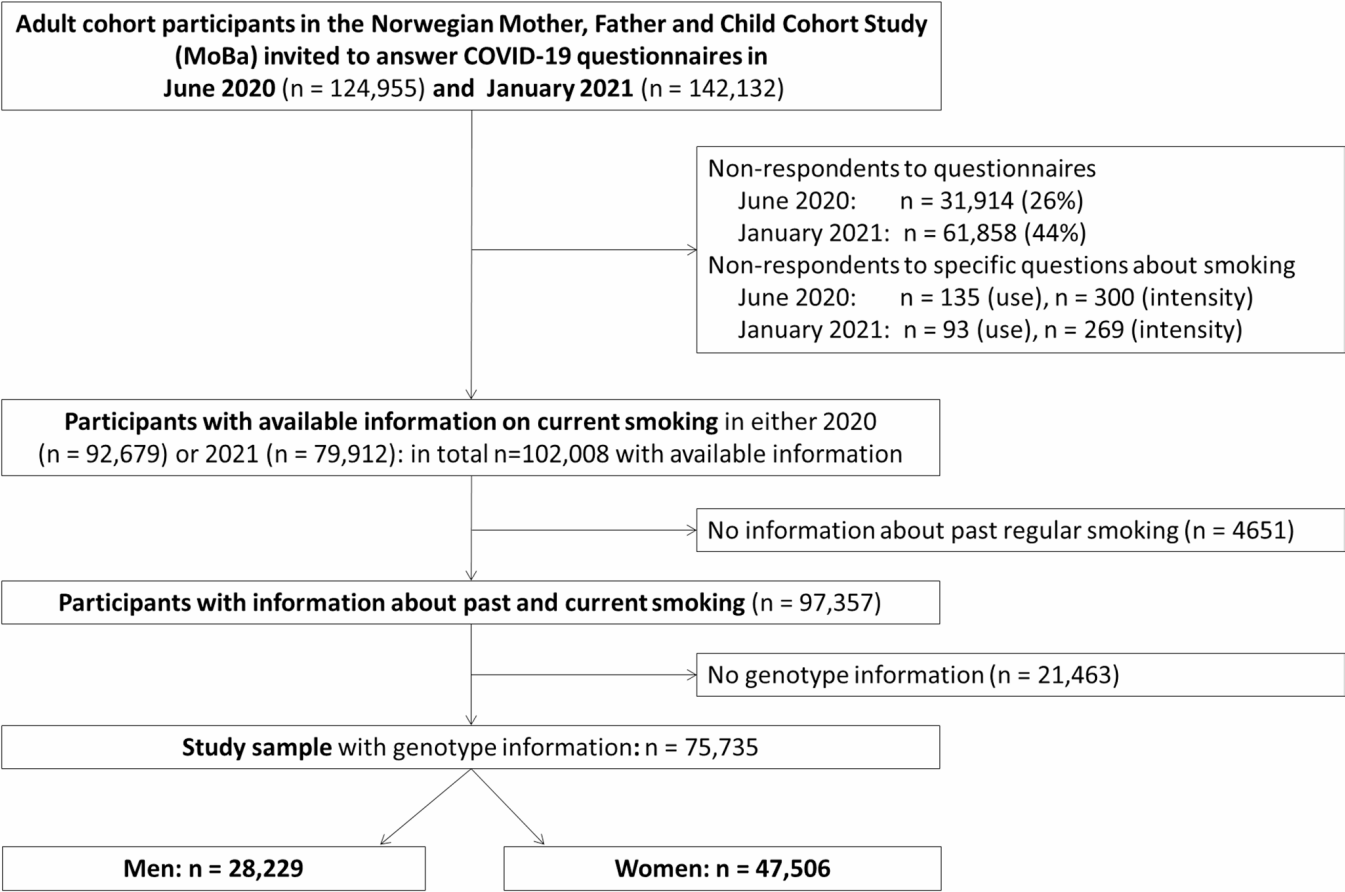
Flow chart for inclusion of participants in the study

Our study included 75,735 subjects with information on current and past smoking and genotype data (Fig. 1). Information about SARS-CoV-2 infection was available for all participants. The study sample consisted of 9% current smokers (10% of women and 7% of men) and 39% ever smokers (current or former, 31% of women and 52% of men) (Table 1). During the study follow-up, 1447 subjects (1.9%) had been diagnosed with COVID-19. On average, men were slightly older than women and had lower education level (Supplementary Table S4). Participants from MoBa who were not included in this study had comparable birth years and BMI to our study sample. However, they were more likely to have a lower level of education (Supplementary Table S5).

**Table 1:** Characteristics of the study sample

	All	Never smoker	Ever smoker (current or past)	Current smoker
	*n* = 75,735	*n* = 46,527	*n* = 29,208	*n* = 6,720
**Gender**,** n (%)**				
Women	47,506 (62.7)	32,851 (70.6)	14,655 (50.2)	4,641 (69.1)
Men	28,229 (37.3)	13,676 (29.4)	14,553 (49.8)	2,079 (30.9)
**Age**^a^, mean ± SD	46.9 (5.2)	46.9 (5.0)	47.0 (5.5)	47.0 (5.5)
**Education level**, n (%)				
< High school	2,388 (3.2)	809 (1.7)	1,579 (5.4)	577 (8.6)
High school	19,934 (26.3)	9,837 (21.1)	10,097 (34.6)	2,780 (41.4)
College, ≤ 4 years	29,440 (38.9)	18,934 (40.7)	10,506 (36.0)	2,206 (32.8)
College, > 4 years	23,973 (31.7)	16,947 (36.4)	7,026 (24.1)	1,157 (17.2)
**No. of cohabitants (mean ± SD)**	3.1 (0.98)	3.2 (0.94)	3.1 (1.0)	2.9 (1.1)
Missing, n (%)	1,746 (2.3)	1,032 (2.2)	714 (2.4)	174 (2.6)
**Region of residence**,** n (%)**				
Oslo metropolitan area/Viken	26,857 (35.5)	16,596 (35.7)	10,261 (35.1)	2,174 (32.4)
Southern Norway	14,414 (19.0)	8,701 (18.7)	5,713 (19.6)	1,401 (20.8)
Western Norway	10,762 (14.2)	6,397 (13.7)	4,365 (14.9)	976 (14.5)
Middle Norway	18,223 (24.1)	11,621 (25.0)	6,602 (22.6)	1,603 (23.9)
Northern Norway	3,760 (5.0)	2,200 (4.7)	1560 (5.3)	393 (5.8)
Missing	1,719 (2.3)	1,012 (2.2)	707 (2.4)	173 (2.6)

^a^ Age at follow-up in 2020

### Smoking initiation (ever smoking)

**Fig. 2:**
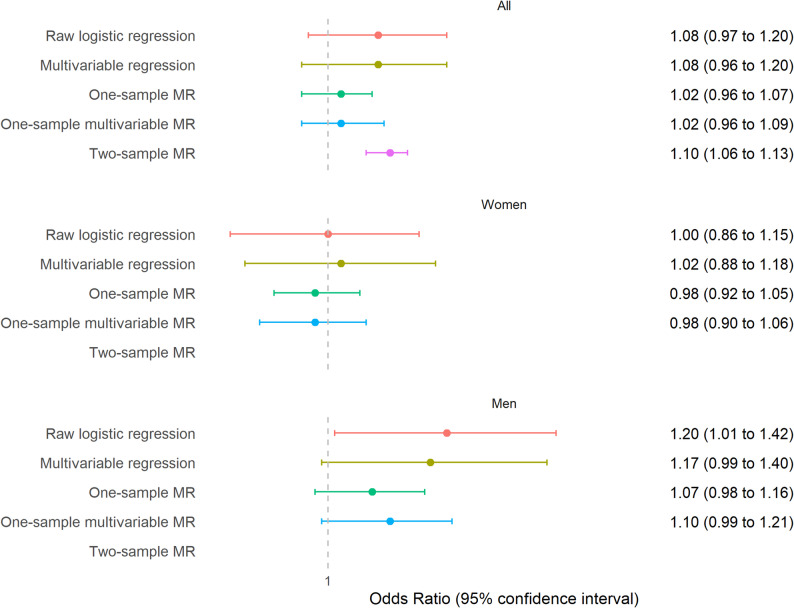
Association between smoking initiation (having ever been a regular smoker) and SARS-CoV-2 infection. Logistic regression compares infection odds between ever and never smokers, while MR analyses assess changes in infection odds per 1 SD increase in the genetic likelihood of smoking initiation

The relationship between having ever been a smoker (smoking initiation) and SARS-CoV-2 infection was unclear when examining both genders together using multivariable regression and one-sample MR (Fig. 2). However, a positive association was observed in the two-sample MR, with an odds ratio (OR) of 1.10 and a 95% confidence interval (CI) ranging from 1.06 to 1.13. Overall, we found no differences in these associations by gender (p-value for interaction: *p* = 0.2 in multivariable analysis and *p* = 0.07 in one-sample MR analysis). No clear associations were found for women across the methods used. For men, the association between having ever been a regular smoker and SARS-CoV-2 infection was OR 1.17, 95% CI 0.99 to 1.40 in the multivariable regression. The strength of this association was comparable to that observed in the two-sample MR (Fig. 2).

When we tested the MR assumption regarding robustness of the genetic instrument, the GRS for smoking initiation was strongly linked to having been a regular smoker in both genders (Supplementary Table S6). However, sensitivity analyses indicated the presence of pleiotropy, as Cochran’s Q and Rücker’s Q′ were both highly significant (*p* < 0.001) (Supplementary Table S7). Multivariable MR findings accounting for education, BMI, and risk-taking behaviors were consistent with those from standard MR analyses. Among men, an association with higher odds of SARS-CoV-2 infection was suggested (OR 1.10, 95% CI 0.99 to 1.21) (Fig. 2), in line with the result from two-sample MR.

### Smoking intensity

**Fig. 3:**
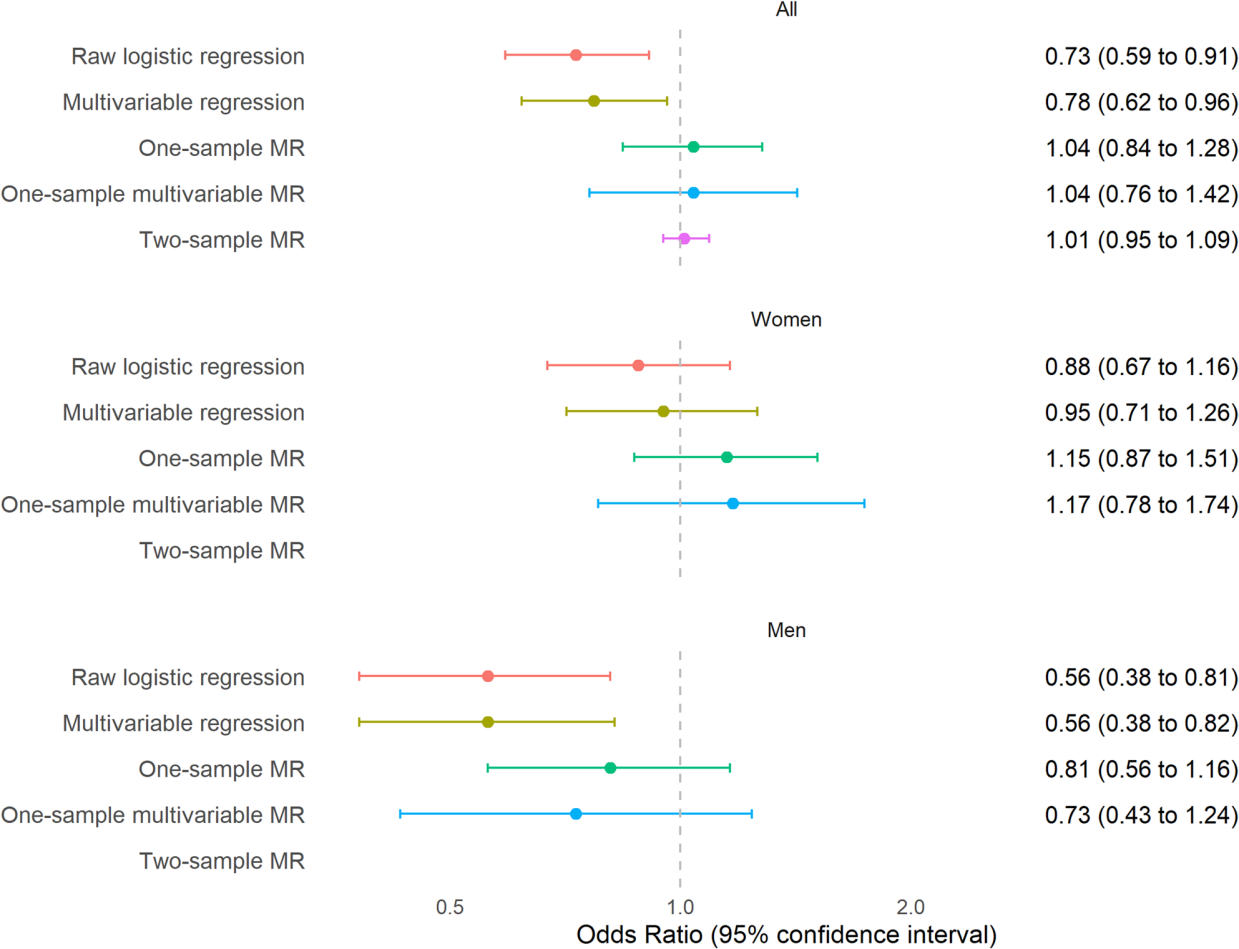
Association between smoking intensity and SARS-CoV-2 infection. Logistic regression shows the odds ratio per 1 SD increase in the number of cigarettes smoked per day, and MR analyses show the odds ratio per 1 SD in the genetically determined number of cigarettes smoked per day

An increase in smoking intensity among current smokers (+ 1 SD in the number of cigarettes smoked per day) was found to be inversely associated with the likelihood of SARS-CoV-2 infection in the standard multivariable regression analysis for the entire population (OR 0.78, 95% CI 0.62 to 0.96), and this association was even stronger when restricted to men (OR 0.56, 95% CI 0.38 to 0.82), p-value for gender interaction: *p* = 0.04). However, this relationship was not observed in MR analyses (Fig. 3) (*p* = 0.54 for gender interaction).

Regarding MR assumptions, the genetic instrument for smoking intensity was robust in women, but weaker in men (Supplementary Table S6). For both genders, the genetic instrument was not associated with SARS-CoV-2 infection among participants who had never reported any current or prior smoking (never smokers) in the sensitivity analyses (Supplementary Table S8). Some heterogeneity between SNPs were indicated (Cochran’s Q p-value = 0.005, Rücker’s Q’ p-value = 0.004), which may reflect pleiotropy (Supplementary Table S7). Since nearly all GRSs for smoking-related traits were associated with years of education and BMI (Supplementary Table S9), we assumed this bias was present for all traits. We performed one-sample multivariable MR analyses while taking into account the genetically determined years of education, BMI values, and the probability of exhibiting risk-taking behaviors [31]. Multivariable MR results were in line with those from standard one-sample MR (Fig. 3).

### Smoking cessation

**Fig. 4:**
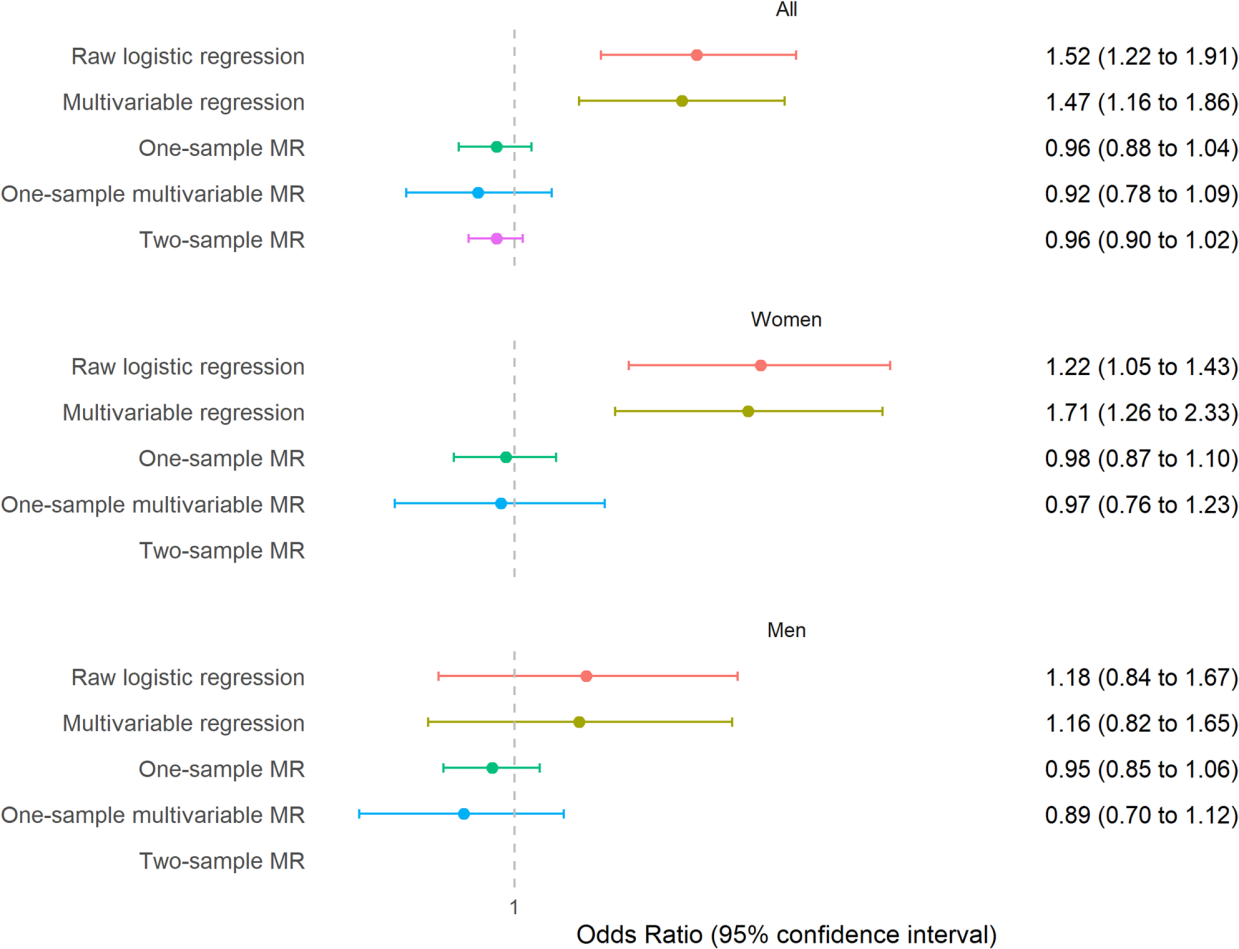
Association between smoking cessation and SARS-CoV-2 infection. Logistic regression compares infection odds between individuals who quit smoking and those who did not, while MR analyses assess changes in infection odds per 1 SD increase in the genetic likelihood of quitting smoking

Quitting smoking among individuals who had ever smoked was associated with increased odds of SARS-CoV-2 infection in the multivariable regression analysis (OR 1.47, 95% CI 1.16 to 1.86). This was particularly noticeable among women (OR 1.71, 95% CI 1.26 to 2.33), with a p-value for gender interaction of *p* = 0.07 in the entire sample. This positive relationship was not observed in MR analyses (Fig. 4). The sensitivity analyses indicated that MR analyses for smoking cessation may be affected by weak instrument bias, as the GRS for this trait was only weakly associated with the exposure in women and not associated with it in men (Supplementary Table S6). Moreover, the GRS was associated with increased odds of SARS-CoV-2 infection in men who never smoked (Supplementary Table S8). In contrast, the estimates for one-sample and two-sample MR were consistent, suggesting that the results may not have been too affected by this bias. Regarding horizontal pleiotropy, there was little evidence of overall heterogeneity, and multivariable one-sample MR findings were consistent with those from standard MR analyses (Supplementary Table 7).

## Discussion

In this study, triangulation of findings from multivariable regression, one-sample MR, and two-sample MR analyses did not provide robust evidence for causal association between smoking and SARS-CoV-2 infection. However, indications of pleiotropy suggest that causal effects cannot be excluded. Specifically, the decreased odds of SARS-CoV-2 infection with increasing smoking intensity observed in the multivariable regression analysis was not replicated in MR analyses. Similarly, the increased odds of infection associated with smoking cessation in the multivariable regression was not replicated in the MR analyses. For smoking initiation, we found indications of a modest increased risk of SARS-CoV-2 infection among men, but in the presence of unexplained horizontal pleiotropy.

The lack of a clear association between smoking intensity and SARS-CoV-2 infection in this study suggests that there is no dose-response relationship between cigarette smoking and being infected with SARS-CoV-2. This finding is not consistent with the study from UK Biobank, where genetically predicted smoking heaviness was associated with 2.5-fold higher odds of confirmed SARS-CoV-2 infection [10]. One explanation for this inconsistency could be related to the degree of smoking heaviness in the two populations, which was higher in the UK Biobank study, where about 71% reported to be smoking at least 10 cigarettes/day (current study: 48%). Notably, while we had information on current smoking collected during the pandemic, this information in the UK Biobank study was collected on average 2.3 years (IQR 0.5 to 9.5 years) prior to the pandemic and may to some degree also reflect previous behavior. In MoBa, participants were recruited as a pregnant couple during 1999 to 2008, almost two decades prior to the COVID-19 pandemic. Therefore, many study subjects likely lived in households with teenagers or young adults in the study period, which may have influenced their smoking behavior, both prior to and during the pandemic.

The suggested, but inconclusive, increased odds of SARS-CoV-2 among men who have ever initiated smoking in this study may reflect an increased risk of SARS-CoV-2 among former smokers [4, 10, 35]. Notably, we did not find significant differences between men and women based on testing of interaction terms. Still, discontinuation of smoking among parents may be different for men and women, for instance if mothers more often quit smoking during pregnancy [36]. We did not examine the influence of smokeless tobacco (snus) use, which is common in this population, especially among men [6]. Although our analyses showed that the association between ever smoking and SARS-CoV-2 was not affected by weak instrument bias or population stratification, sensitivity analyses indicated presence of pleiotropy. The sources of horizontal pleiotropy that we corrected for in our multivariable MR analysis (educational level, BMI and risk-taking behaviors) did not weaken the association. Notably, the direction of associations for men was similar across methods, indicating that the observed association may still be causal. The inverse association between smoking and SARS-CoV-2 infection reported in several observational studies, sometimes referred to as the “smoker’s paradox” [37], has been attributed to mechanisms such as altered ACE2 expression [38]. Our findings do not support these hypotheses.

We hypothesized that the observed association between current smoking and SARS-CoV-2 infection observed in MR studies may partly be explained by a shared genetic basis between behavioral aspects (such as risk-taking behavior) and smoking initiation and continuation [19]. However, correcting for risk-taking behavior in the one-sample multivariable MR analysis did not change our estimates. One explanation may be that the genetic instrument applied did not appropriately capture risk-taking behavior in this population. We did not have observational data to verify this assumption. It is also possible that the genetic variants related to risk-taking behavior may be associated with trying out cigarette smoking rather than the transition to regular use [19], which was the smoking behavior studied here. If so, risk-taking behavior may not be a common trait among current smokers, and thus not an important confounder.

A main strength of our study is that we could examine gender-specific associations between smoking behavior assessed at multiple time points and a COVID-19 diagnosis using a large population-based cohort with information on all positive SARS-CoV-2 PCR tests. During the study period, PCR testing was universally accessible in Norway, and reporting of positive results to the national registry was mandatory. In a previous study from the same sample, we did not find evidence that testing was less common among tobacco users compared to non-users, but rather slightly higher [6]. Overall, the low number of infected subjects in this study reflects the epidemiological situation in Norway in this period, and we expect that the likelihood of misclassification of positive test results is low. The regular follow-up of the cohort during the pandemic enabled us to define the study period to mainly cover the pre- and early vaccination period of the pandemic in Norway. Thus, we could investigate these associations without intermediate effects of vaccine hesitancy and/or effectiveness.

Our study also has some important limitations. First, the genetic instruments used for measuring, as smoking cessation, as well as for smoking intensity in men, were weak. Weak instruments biases the estimates away from the null in one-sample MR and towards the null in two-sample MR [28]. Any relationship between these traits and infection observed in one-sample MR but not in two-sample MR could therefore be attributed to weak instrument bias. However, we did not find any such association. Further, participants may have changed their smoking behavior during the pandemic, which may result in exposure misclassification of the observed trait. Second, although we expect that the misclassification of SARS-CoV-2 infection is relatively low, it is likely that some infections, especially asymptomatic, may have gone undetected due to lack of testing. Third, infection prevalence was low during the study period, which, in combination with weak instruments, likely reduced statistical power. Fourth, our results might be influenced by selection. MoBa participants are likely to have a higher educational level than the general population, and smokers are underrepresented [39]. Participants in MoBa are parents to children born in 1999 to 2009. Because questions on smoking were asked in the context of parenthood, where smoking is discouraged, reporting of smoking may be more influenced by social desirability and recall than in a general adult population, and thus potentially more prone to misclassification bias. These factors could potentially reduce the external validity of our findings. Fifth, the generalizability of our findings is limited by our study population, which consists of adult women and men of Northern European ancestry.

Lastly, there are further limitations to the interpretation of our MR findings, especially related to horizontal pleiotropy. The genetic instruments for smoking traits may affect infection risk through multiple biological and behavioral pathways. We examined some pleiotropic pathways, including BMI, educational attainment and risk-taking behavior. However, other potential pleiotropic pathways, including differences in testing and other health-related behavior, occupational factors, and comorbidities, were not explored in this study. Also, SARS-CoV-2 infection is a transient phenotype, and the risk of being infected varies both by the individual susceptibility and by the time- and location-specific epidemiological situation. The underlying assumptions in MR analysis may therefore be particularly difficult to meet for acute infectious outcomes. Although the MR approach still can provide useful evidence on the potential causal role of smoking on infection risk, the pandemic setting warrants that our findings should be interpreted with particular caution.

In conclusion, the lack of robust evidence of an association between smoking intensity and SARS-CoV-2 infection does not support previous observational studies showing reduced risk among current smokers. Still, uncertainties related to the underlying MR assumptions imply that a causal effect cannot be ruled out. By highlighting these methodological challenges, our study may help explain inconsistencies between observational and MR results. It also supports a careful interpretation of MR findings of smoking in the context of a pandemic.

## Supplementary Information

Below is the link to the electronic supplementary material.

Supplementary Material 1

Supplementary Material 2

Supplementary Material 3

## Data Availability

Data from the Norwegian Mother, Father and Child Cohort Study and the Medical Birth Registry of Norway used in this study are managed by the national health register holders in Norway (Norwegian Institute of Public Health) and can be made available to researchers, provided approval from the Regional Committees for Medical and Health Research Ethics (REC), compliance with the EU General Data Protection Regulation (GDPR) and approval from the data owners. The consent given by the participants does not open for storage of data on an individual level in repositories or journals. Researchers who want access to data sets for replication should apply through helsedata.no. Access to data sets requires approval from The Regional Committee for Medical and Health Research Ethics in Norway and an agreement with MoBa.

## Abbreviations

ACE2: Angiotensin-converting enzyme 2
BMI: Body mass index
CI: Confidence interval
GWAS: Genome-wide association study
GRS: Genetic risk score, polygenic risk score
IQR: Interquartile range (P25 to P75)
IVW: Inverse variance weighted
LD: Linkage disequilibrium
MR: Mendelian randomization
MoBa: Norwegian Mother, Father, and Child Cohort Study
MSIS: Norwegian Surveillance System for Communicable Diseases
OR: Odds ratio
PCR: Polymerase chain reaction
SD: Standard deviation
SNP: Single nucleotide polymorphism
STROBE: Strengthening the Reporting of Observational Studies in Epidemiology
