# Supplementary material for: Cigarette smoking and SARS-CoV-2 infection: multivariable regression and Mendelian randomization analyses in the Norwegian Mother, Father and Child Cohort Study
Source: BMC Infect Dis. 2026 Feb 4;26:509. doi: 10.1186/s12879-026-12750-8 (PMC12964713; doi:10.1186/s12879-026-12750-8)
Supplement: Supplementary file 3 — Supplementary Material 3 [file 12879_2026_12750_MOESM3_ESM.docx]

**SUPPLEMENTARY MATERIAL**

**Smoking and SARS-CoV-2 infection: One- and two-sample Mendelian randomization analyses based on the Norwegian Mother, Father and Child Cohort Study**

Ida Henriette Caspersen (ida.henriette.caspersen@fhi.no), Álvaro Hernáez, Sebastián Peña, Ahmed Nabil Shaaban, Maria Christine Magnus, Sakari Karvonen, Maria Rosaria Galanti, Per Magnus

[**Supplementary Table S1**. Genetic instruments for smoking initiation 2](#_Toc160808645)

[**Supplementary Table S2.** Genetic instruments for smoking intensity (cigarettes smoked per day) 8](#_Toc160808646)

[**Supplementary Table S3.** Genetic instruments for smoking cessation 9](#_Toc160808647)

[**Supplementary Table S4.** Sex-stratified description of the population 10](#_Toc160808648)

[**Supplementary Table S5**. Comparison of MoBa participants included and not included in our analyses 11](#_Toc160808649)

[**Supplementary Table S6**. Robustness of genetic instruments 12](#_Toc160808650)

[**Supplementary Table S7.** Two-sample Mendelian randomization analyses: main analysis (inverse variance weighted method) and sensitivity analyses (MR-Egger, weighted median, weighted mode) to validate the lack of horizontal pleiotropy 13](#_Toc160808651)

[**Supplementary Table S8**. Associations of GRSs of smoking-related traits with odds of COVID-19 infection in never smokers (non-relevance sensitivity analyses) 14](#_Toc160808652)

[**Supplementary Table S9**. Associations between genetic risk scores for smoking-related traits and COVID-19 infection risk factors with information available in MoBa 15](#_Toc160808653)

# **Supplementary Table S1**. Genetic instruments for smoking initiation

| **RSID** | **Chrom.** | **Position** | **Reference**  **Allele** | **Effect**  **Allele** | **Effect**  **Allele**  **Freq.** | **Beta** | **SE** | **p-value** | **One-**  **sample**  **MR** | **Two-**  **sample**  **MR** |
| --- | --- | --- | --- | --- | --- | --- | --- | --- | --- | --- |
| rs12130857 | 1 | 7791461 | G | A | 0.325 | -0.018 | 0.0027 | 3.65E-11 | Yes | Yes |
| rs301807 | 1 | 8484823 | A | G | 0.570 | 0.018 | 0.0026 | 2.50E-12 | Yes | Yes |
| rs3820277 | 1 | 18436657 | G | T | 0.526 | -0.019 | 0.0026 | 1.57E-13 | Yes | Yes |
| rs1889571 | 1 | 32195819 | T | G | 0.131 | 0.022 | 0.0038 | 4.19E-09 | Yes | Yes |
| rs10914684 | 1 | 33795572 | G | A | 0.324 | -0.016 | 0.0027 | 6.32E-09 | Yes | Yes |
| rs2637869 | 1 | 38757237 | G | A | 0.297 | 0.018 | 0.0028 | 6.54E-11 | Yes | Yes |
| rs12755632 | 1 | 41776623 | A | G | 0.316 | -0.015 | 0.0027 | 1.93E-08 | Yes | Yes |
| rs951740 | 1 | 44011737 | G | A | 0.625 | 0.030 | 0.0026 | 3.82E-29 | Yes | Yes |
| rs925524 | 1 | 46496709 | A | G | 0.710 | 0.016 | 0.0028 | 2.94E-08 | Yes | Yes |
| rs12022778 | 1 | 50603995 | A | C | 0.202 | 0.027 | 0.0032 | 3.18E-17 | Yes | Yes |
| rs11587399 | 1 | 50861071 | A | T | 0.221 | -0.018 | 0.0031 | 7.25E-09 | Yes | Yes |
| rs4912332 | 1 | 58815243 | C | T | 0.491 | 0.014 | 0.0025 | 2.94E-08 | Yes | Yes |
| rs1937443 | 1 | 66469643 | C | G | 0.563 | 0.020 | 0.0026 | 1.79E-15 | Yes | No |
| rs1022528 | 1 | 71490122 | G | A | 0.344 | 0.017 | 0.0027 | 8.48E-11 | Yes | Yes |
| rs12740789 | 1 | 72752073 | G | A | 0.178 | -0.028 | 0.0033 | 1.18E-17 | Yes | Yes |
| rs80054503 | 1 | 72900406 | T | C | 0.116 | -0.024 | 0.0041 | 3.10E-09 | No | No |
| rs10789369 | 1 | 73824909 | A | G | 0.615 | -0.023 | 0.0026 | 3.39E-19 | Yes | Yes |
| rs1514176 | 1 | 74991596 | G | A | 0.580 | -0.019 | 0.0026 | 7.67E-14 | Yes | Yes |
| rs10873871 | 1 | 76689019 | A | G | 0.207 | 0.017 | 0.0031 | 2.82E-08 | Yes | Yes |
| rs11162019 | 1 | 87913176 | C | T | 0.363 | -0.015 | 0.0026 | 5.06E-09 | Yes | Yes |
| rs1008078 | 1 | 91189731 | C | T | 0.402 | 0.023 | 0.0026 | 1.63E-18 | Yes | Yes |
| rs1935571 | 1 | 96414335 | T | G | 0.480 | -0.016 | 0.0026 | 6.99E-10 | Yes | Yes |
| rs12027999 | 1 | 154206358 | T | C | 0.120 | -0.024 | 0.0039 | 5.33E-10 | Yes | Yes |
| rs45444697 | 1 | 155034632 | C | G | 0.212 | 0.020 | 0.0031 | 2.72E-10 | Yes | Yes |
| rs2901785 | 1 | 174104743 | G | A | 0.446 | -0.017 | 0.0026 | 1.47E-11 | Yes | Yes |
| rs147052174 | 1 | 179783167 | G | T | 0.017 | 0.062 | 0.0098 | 2.30E-10 | Yes | Yes |
| rs35656245 | 1 | 190957480 | G | A | 0.276 | 0.016 | 0.0029 | 2.23E-08 | Yes | Yes |
| rs12739243 | 1 | 210302043 | T | C | 0.221 | -0.021 | 0.0031 | 4.45E-12 | Yes | Yes |
| rs12563365 | 1 | 236872829 | G | A | 0.556 | 0.017 | 0.0026 | 1.05E-10 | Yes | Yes |
| rs876793 | 1 | 237852083 | T | C | 0.349 | -0.018 | 0.0027 | 5.69E-11 | Yes | Yes |
| rs114976176 | 2 | 264621 | A | C | 0.352 | -0.016 | 0.0027 | 6.04E-09 | Yes | Yes |
| rs62106258 | 2 | 417167 | T | C | 0.047 | -0.045 | 0.0060 | 3.33E-14 | No | Yes |
| rs6731872 | 2 | 624205 | T | G | 0.826 | 0.032 | 0.0034 | 5.35E-21 | Yes | Yes |
| rs1022376 | 2 | 22067213 | T | C | 0.516 | -0.015 | 0.0026 | 1.66E-08 | Yes | Yes |
| rs61533748 | 2 | 22582968 | T | C | 0.384 | 0.017 | 0.0026 | 2.82E-11 | Yes | Yes |
| rs72790288 | 2 | 29513404 | G | A | 0.028 | -0.046 | 0.0077 | 3.28E-09 | Yes | Yes |
| rs2710634 | 2 | 32808804 | T | C | 0.521 | -0.018 | 0.0026 | 3.36E-12 | Yes | Yes |
| rs62137126 | 2 | 44250149 | A | G | 0.121 | -0.024 | 0.0039 | 1.31E-09 | Yes | Yes |
| rs1004787 | 2 | 45159091 | G | A | 0.552 | 0.028 | 0.0026 | 1.11E-28 | Yes | Yes |
| rs7598402 | 2 | 50735943 | C | G | 0.492 | -0.015 | 0.0025 | 7.38E-09 | Yes | No |
| rs10490159 | 2 | 51341259 | C | T | 0.394 | 0.017 | 0.0026 | 3.86E-11 | Yes | Yes |
| rs1518393 | 2 | 58171220 | A | C | 0.619 | 0.017 | 0.0026 | 1.30E-10 | Yes | Yes |
| rs17616642 | 2 | 59022210 | A | G | 0.247 | -0.017 | 0.0030 | 2.10E-08 | Yes | Yes |
| rs6730325 | 2 | 59315828 | G | A | 0.610 | -0.015 | 0.0026 | 2.10E-08 | Yes | Yes |
| rs2539706 | 2 | 59819545 | G | A | 0.530 | 0.016 | 0.0026 | 1.95E-10 | Yes | Yes |
| rs7585579 | 2 | 60024857 | C | G | 0.499 | 0.020 | 0.0026 | 5.48E-15 | Yes | No |
| rs1863161 | 2 | 60139524 | G | A | 0.561 | 0.015 | 0.0026 | 2.34E-09 | Yes | Yes |
| rs359247 | 2 | 60477052 | A | T | 0.639 | 0.022 | 0.0027 | 9.89E-17 | Yes | Yes |
| rs62180324 | 2 | 63416606 | G | A | 0.212 | -0.020 | 0.0031 | 3.91E-10 | Yes | Yes |
| rs6750107 | 2 | 80748807 | G | A | 0.387 | 0.015 | 0.0026 | 2.60E-08 | Yes | Yes |
| rs12714017 | 2 | 80999398 | T | C | 0.511 | 0.015 | 0.0026 | 3.65E-09 | Yes | Yes |
| rs56208390 | 2 | 83247997 | A | G | 0.123 | 0.022 | 0.0039 | 2.68E-08 | Yes | Yes |
| rs11692435 | 2 | 98275354 | G | A | 0.085 | 0.025 | 0.0046 | 4.47E-08 | Yes | Yes |
| rs13392222 | 2 | 100672408 | A | C | 0.139 | -0.023 | 0.0037 | 1.93E-10 | Yes | Yes |
| rs1901477 | 2 | 104126983 | A | G | 0.511 | 0.030 | 0.0026 | 2.07E-31 | Yes | Yes |
| rs11889814 | 2 | 104432494 | A | C | 0.128 | -0.021 | 0.0038 | 3.44E-08 | Yes | Yes |
| rs3811038 | 2 | 113240183 | T | C | 0.279 | 0.019 | 0.0028 | 1.58E-11 | Yes | Yes |
| rs75210106 | 2 | 113246436 | C | T | 0.177 | -0.019 | 0.0033 | 2.33E-08 | Yes | Yes |
| rs34399632 | 2 | 137571174 | A | G | 0.232 | 0.019 | 0.0030 | 1.46E-10 | Yes | Yes |
| rs74697736 | 2 | 145412271 | G | A | 0.287 | 0.022 | 0.0028 | 2.43E-15 | Yes | Yes |
| rs6756212 | 2 | 146140132 | C | T | 0.535 | -0.034 | 0.0026 | 3.49E-40 | Yes | Yes |
| rs3076896 | 2 | 146283610 | G | A | 0.390 | 0.023 | 0.0028 | 1.99E-16 | No | No |
| rs16826827 | 2 | 147825689 | T | C | 0.124 | -0.022 | 0.0039 | 9.17E-09 | Yes | Yes |
| rs1445649 | 2 | 155682556 | T | C | 0.538 | 0.021 | 0.0026 | 8.48E-16 | Yes | Yes |
| rs1722666 | 2 | 161816880 | C | T | 0.732 | 0.016 | 0.0029 | 2.17E-08 | Yes | Yes |
| rs11678980 | 2 | 162101261 | G | A | 0.450 | 0.018 | 0.0026 | 5.19E-12 | No | Yes |
| rs12474587 | 2 | 162802993 | G | T | 0.429 | 0.024 | 0.0026 | 4.83E-21 | Yes | Yes |
| rs357304 | 2 | 164862639 | T | C | 0.727 | 0.017 | 0.0029 | 5.40E-09 | Yes | Yes |
| rs13007361 | 2 | 166250244 | G | A | 0.208 | 0.018 | 0.0031 | 2.29E-08 | Yes | Yes |
| rs7600835 | 2 | 172521827 | G | A | 0.342 | -0.015 | 0.0027 | 1.80E-08 | Yes | Yes |
| rs6750529 | 2 | 182027603 | C | T | 0.744 | 0.020 | 0.0029 | 9.26E-12 | Yes | Yes |
| rs17229285 | 2 | 199523122 | C | T | 0.505 | -0.015 | 0.0025 | 1.27E-09 | Yes | Yes |
| rs3115418 | 2 | 200936399 | T | C | 0.454 | -0.014 | 0.0026 | 2.79E-08 | Yes | Yes |
| rs62193862 | 2 | 202843875 | G | A | 0.100 | 0.024 | 0.0042 | 1.99E-08 | Yes | Yes |
| rs4674916 | 2 | 225365635 | C | A | 0.328 | -0.018 | 0.0027 | 3.06E-11 | Yes | Yes |
| rs4674993 | 2 | 226332033 | A | G | 0.200 | -0.024 | 0.0032 | 4.85E-14 | Yes | Yes |
| rs11713899 | 3 | 2365026 | A | C | 0.171 | 0.019 | 0.0034 | 3.15E-08 | Yes | Yes |
| rs748832 | 3 | 16851202 | A | G | 0.371 | 0.017 | 0.0026 | 6.60E-11 | Yes | Yes |
| rs10446419 | 3 | 25725501 | A | G | 0.207 | -0.020 | 0.0031 | 5.05E-10 | No | Yes |
| rs13319205 | 3 | 47800216 | T | A | 0.290 | 0.017 | 0.0028 | 3.77E-09 | Yes | Yes |
| rs3172494 | 3 | 48731487 | G | T | 0.115 | -0.029 | 0.0040 | 3.40E-13 | Yes | Yes |
| rs2526390 | 3 | 50192760 | C | T | 0.334 | 0.020 | 0.0027 | 3.62E-14 | Yes | Yes |
| rs2276825 | 3 | 52886605 | T | C | 0.245 | 0.019 | 0.0030 | 1.89E-10 | Yes | Yes |
| rs2306866 | 3 | 53766212 | A | T | 0.614 | -0.017 | 0.0026 | 1.89E-10 | Yes | Yes |
| rs73831818 | 3 | 55988394 | A | G | 0.057 | 0.032 | 0.0055 | 5.46E-09 | Yes | Yes |
| rs1910236 | 3 | 59434420 | G | A | 0.469 | 0.015 | 0.0026 | 9.91E-09 | No | Yes |
| rs7640107 | 3 | 59966156 | C | T | 0.431 | -0.014 | 0.0026 | 3.46E-08 | Yes | Yes |
| rs2734390 | 3 | 60459291 | A | G | 0.372 | 0.015 | 0.0026 | 2.09E-08 | Yes | Yes |
| rs221988 | 3 | 64234307 | A | C | 0.384 | -0.015 | 0.0026 | 1.43E-08 | Yes | Yes |
| rs2196356 | 3 | 70890288 | G | C | 0.289 | -0.019 | 0.0028 | 2.45E-11 | Yes | Yes |
| rs11128203 | 3 | 71064431 | T | A | 0.530 | 0.020 | 0.0026 | 1.29E-15 | No | No |
| rs62246017 | 3 | 71483084 | G | A | 0.323 | -0.016 | 0.0027 | 3.03E-09 | Yes | Yes |
| rs4543050 | 3 | 74954560 | A | T | 0.816 | 0.022 | 0.0033 | 1.45E-11 | Yes | Yes |
| rs6782116 | 3 | 77176032 | C | T | 0.415 | -0.015 | 0.0026 | 1.46E-08 | Yes | Yes |
| rs13066050 | 3 | 81325861 | C | T | 0.208 | 0.019 | 0.0031 | 1.93E-09 | Yes | Yes |
| rs12633090 | 3 | 83241365 | G | C | 0.182 | -0.023 | 0.0033 | 3.16E-12 | Yes | Yes |
| rs1549979 | 3 | 85460131 | C | T | 0.615 | -0.025 | 0.0026 | 8.80E-21 | Yes | Yes |
| rs74664784 | 3 | 85475292 | T | C | 0.376 | -0.020 | 0.0028 | 9.34E-13 | No | Yes |
| rs57153235 | 3 | 85902536 | T | G | 0.318 | -0.019 | 0.0027 | 1.56E-12 | Yes | Yes |
| rs6437769 | 3 | 107997514 | C | T | 0.581 | 0.014 | 0.0026 | 3.74E-08 | Yes | Yes |
| rs9288999 | 3 | 114147927 | G | A | 0.735 | 0.017 | 0.0029 | 1.50E-09 | Yes | Yes |
| rs6438436 | 3 | 117822149 | C | T | 0.816 | 0.025 | 0.0033 | 5.33E-14 | Yes | Yes |
| rs12053870 | 3 | 118302515 | T | G | 0.542 | 0.016 | 0.0026 | 1.02E-09 | Yes | Yes |
| rs9826984 | 3 | 131945722 | G | A | 0.542 | -0.014 | 0.0026 | 3.87E-08 | Yes | Yes |
| rs2279829 | 3 | 147106319 | C | T | 0.216 | -0.017 | 0.0031 | 2.05E-08 | Yes | Yes |
| rs2319545 | 3 | 147719648 | C | A | 0.149 | 0.023 | 0.0036 | 8.30E-11 | Yes | Yes |
| rs10935779 | 3 | 149543102 | C | T | 0.415 | -0.014 | 0.0026 | 2.95E-08 | Yes | Yes |
| rs963354 | 3 | 157393770 | C | A | 0.687 | 0.015 | 0.0027 | 4.21E-08 | Yes | Yes |
| rs1714521 | 3 | 158284861 | A | C | 0.411 | -0.016 | 0.0026 | 3.07E-10 | Yes | Yes |
| rs1449012 | 3 | 159048333 | C | T | 0.463 | -0.015 | 0.0026 | 1.77E-09 | Yes | Yes |
| rs9850597 | 3 | 161761866 | G | A | 0.816 | -0.019 | 0.0033 | 1.65E-08 | Yes | Yes |
| rs1187820 | 3 | 173072584 | C | T | 0.439 | -0.014 | 0.0026 | 2.69E-08 | Yes | Yes |
| rs16828799 | 3 | 173353739 | G | T | 0.156 | 0.020 | 0.0035 | 1.83E-08 | Yes | Yes |
| rs9841807 | 3 | 175718927 | C | T | 0.273 | 0.016 | 0.0029 | 1.35E-08 | Yes | Yes |
| rs7631379 | 3 | 181409057 | T | C | 0.206 | 0.021 | 0.0032 | 3.94E-11 | Yes | Yes |
| rs4140932 | 4 | 15458598 | T | A | 0.431 | -0.014 | 0.0026 | 4.89E-08 | Yes | No |
| rs12642744 | 4 | 28027176 | G | T | 0.744 | -0.017 | 0.0030 | 2.82E-08 | Yes | Yes |
| rs59537158 | 4 | 28246049 | C | T | 0.214 | 0.022 | 0.0031 | 4.62E-13 | Yes | Yes |
| rs1389171 | 4 | 28822284 | T | A | 0.241 | -0.017 | 0.0030 | 4.45E-09 | Yes | Yes |
| rs55944129 | 4 | 29082156 | T | C | 0.267 | -0.018 | 0.0029 | 1.06E-09 | Yes | Yes |
| rs58400863 | 4 | 31184484 | G | A | 0.347 | -0.020 | 0.0027 | 4.89E-14 | Yes | Yes |
| rs7657022 | 4 | 35501032 | A | G | 0.489 | 0.018 | 0.0025 | 7.34E-13 | Yes | Yes |
| rs55900829 | 4 | 35514712 | A | T | 0.335 | 0.019 | 0.0028 | 5.63E-12 | No | No |
| rs112725451 | 4 | 68017710 | C | T | 0.169 | 0.026 | 0.0034 | 1.65E-14 | Yes | Yes |
| rs1160685 | 4 | 94052854 | C | G | 0.450 | 0.015 | 0.0026 | 2.31E-09 | Yes | No |
| rs1435479 | 4 | 94550450 | G | T | 0.287 | 0.016 | 0.0028 | 5.68E-09 | Yes | Yes |
| rs3934797 | 4 | 112467612 | G | A | 0.182 | -0.021 | 0.0033 | 1.12E-10 | Yes | Yes |
| rs71602617 | 4 | 136406155 | C | T | 0.216 | -0.018 | 0.0032 | 2.10E-08 | Yes | Yes |
| rs7696257 | 4 | 137474783 | G | A | 0.366 | 0.015 | 0.0026 | 6.78E-09 | Yes | Yes |
| rs13109980 | 4 | 140886963 | G | A | 0.326 | -0.022 | 0.0027 | 3.37E-16 | Yes | Yes |
| rs1116690 | 4 | 143510148 | A | G | 0.742 | 0.016 | 0.0029 | 2.16E-08 | Yes | Yes |
| rs13110073 | 4 | 147797913 | T | C | 0.395 | -0.025 | 0.0026 | 3.24E-21 | Yes | Yes |
| rs28717373 | 4 | 147985231 | C | T | 0.356 | -0.016 | 0.0027 | 6.16E-10 | Yes | Yes |
| rs62340589 | 4 | 176875795 | G | C | 0.201 | 0.017 | 0.0032 | 4.31E-08 | Yes | Yes |
| rs12517438 | 5 | 30842054 | T | G | 0.538 | 0.015 | 0.0026 | 1.89E-09 | Yes | Yes |
| rs35375873 | 5 | 43190647 | G | C | 0.110 | -0.027 | 0.0041 | 3.29E-11 | No | Yes |
| rs986714 | 5 | 50821338 | A | T | 0.445 | -0.016 | 0.0026 | 4.13E-10 | Yes | No |
| rs71592686 | 5 | 60121271 | T | C | 0.274 | 0.021 | 0.0029 | 3.85E-13 | Yes | Yes |
| rs2028269 | 5 | 79308315 | G | A | 0.399 | 0.016 | 0.0026 | 5.19E-10 | Yes | Yes |
| rs6874731 | 5 | 80263865 | T | G | 0.484 | 0.015 | 0.0025 | 1.83E-09 | Yes | Yes |
| rs6452785 | 5 | 87685500 | C | T | 0.474 | -0.027 | 0.0026 | 4.69E-26 | Yes | Yes |
| rs10805858 | 5 | 88873832 | A | T | 0.335 | 0.018 | 0.0027 | 1.88E-11 | Yes | Yes |
| rs181508347 | 5 | 91366274 | T | G | 0.010 | 0.081 | 0.0130 | 4.95E-10 | Yes | Yes |
| rs42417 | 5 | 94198290 | C | T | 0.691 | 0.017 | 0.0028 | 8.27E-10 | Yes | Yes |
| rs72780746 | 5 | 103929588 | T | C | 0.173 | -0.026 | 0.0034 | 2.05E-14 | Yes | Yes |
| rs10060196 | 5 | 106455988 | C | A | 0.581 | 0.018 | 0.0026 | 1.29E-12 | Yes | Yes |
| rs72789626 | 5 | 106825618 | T | A | 0.136 | -0.026 | 0.0037 | 5.13E-12 | Yes | Yes |
| rs17165769 | 5 | 107365642 | A | G | 0.395 | 0.016 | 0.0026 | 9.56E-10 | Yes | Yes |
| rs329124 | 5 | 133865452 | A | G | 0.428 | -0.016 | 0.0026 | 1.96E-10 | Yes | Yes |
| rs1385108 | 5 | 154839646 | C | T | 0.239 | 0.019 | 0.0030 | 3.84E-10 | Yes | Yes |
| rs1173461 | 5 | 157707571 | C | T | 0.327 | 0.017 | 0.0027 | 9.51E-10 | Yes | Yes |
| rs11956866 | 5 | 161018271 | T | G | 0.567 | -0.015 | 0.0026 | 7.82E-09 | Yes | Yes |
| rs3909281 | 5 | 165096435 | T | G | 0.536 | 0.021 | 0.0026 | 1.62E-16 | Yes | Yes |
| rs3843905 | 5 | 165427280 | C | T | 0.403 | -0.015 | 0.0026 | 5.41E-09 | Yes | Yes |
| rs79476395 | 5 | 166063680 | A | G | 0.073 | 0.033 | 0.0049 | 1.04E-11 | Yes | Yes |
| rs6890961 | 5 | 166778503 | C | T | 0.624 | -0.019 | 0.0026 | 2.13E-13 | Yes | Yes |
| rs4044321 | 5 | 166989513 | A | G | 0.644 | -0.023 | 0.0027 | 1.75E-17 | Yes | Yes |
| rs2173019 | 5 | 167614971 | T | A | 0.177 | 0.028 | 0.0033 | 2.98E-17 | No | Yes |
| rs10042827 | 5 | 170299916 | T | C | 0.681 | 0.017 | 0.0027 | 9.41E-10 | Yes | Yes |
| rs359431 | 5 | 173288534 | C | T | 0.560 | -0.014 | 0.0026 | 3.16E-08 | Yes | Yes |
| rs1059490 | 6 | 26171250 | T | C | 0.367 | -0.019 | 0.0026 | 2.16E-12 | Yes | Yes |
| rs6932350 | 6 | 26571629 | T | A | 0.455 | 0.015 | 0.0026 | 5.13E-09 | Yes | No |
| rs1150668 | 6 | 28129789 | T | G | 0.419 | -0.019 | 0.0026 | 8.54E-13 | Yes | Yes |
| rs1632941 | 6 | 29796685 | T | C | 0.460 | -0.016 | 0.0026 | 6.67E-10 | Yes | Yes |
| rs3218116 | 6 | 41901763 | C | T | 0.256 | -0.020 | 0.0029 | 1.05E-11 | Yes | Yes |
| rs160631 | 6 | 52895230 | T | G | 0.731 | -0.017 | 0.0029 | 1.87E-09 | Yes | Yes |
| rs7743165 | 6 | 67521222 | T | G | 0.495 | 0.019 | 0.0025 | 4.15E-14 | Yes | Yes |
| rs79180767 | 6 | 67540984 | C | T | 0.253 | 0.020 | 0.0029 | 7.00E-12 | No | No |
| rs10945141 | 6 | 69470709 | G | A | 0.263 | 0.018 | 0.0029 | 3.59E-10 | Yes | Yes |
| rs17554906 | 6 | 92226609 | G | C | 0.444 | 0.014 | 0.0026 | 3.14E-08 | Yes | No |
| rs619087 | 6 | 94175279 | A | G | 0.422 | 0.014 | 0.0026 | 3.10E-08 | Yes | Yes |
| rs6568832 | 6 | 97702876 | G | A | 0.754 | 0.019 | 0.0030 | 1.74E-10 | Yes | Yes |
| rs12195240 | 6 | 98636905 | G | A | 0.285 | 0.025 | 0.0028 | 1.08E-18 | Yes | Yes |
| rs6936160 | 6 | 100347745 | C | T | 0.698 | 0.020 | 0.0028 | 4.20E-13 | Yes | Yes |
| rs12530388 | 6 | 101329173 | A | C | 0.511 | -0.018 | 0.0025 | 5.83E-13 | Yes | Yes |
| rs3800227 | 6 | 108994161 | A | G | 0.742 | 0.017 | 0.0029 | 3.64E-09 | Yes | Yes |
| rs118202 | 6 | 111658371 | G | T | 0.812 | -0.037 | 0.0033 | 1.90E-29 | Yes | Yes |
| rs73008357 | 6 | 156431856 | A | C | 0.121 | -0.022 | 0.0040 | 2.44E-08 | Yes | Yes |
| rs9331343 | 6 | 157738258 | T | C | 0.568 | -0.014 | 0.0026 | 3.90E-08 | No | Yes |
| rs10698713 | 6 | 158882320 | G | A | 0.054 | -0.034 | 0.0056 | 2.38E-09 | Yes | Yes |
| rs1737329 | 6 | 163807748 | C | G | 0.742 | 0.017 | 0.0029 | 5.08E-09 | Yes | Yes |
| rs10272990 | 7 | 1703675 | T | C | 0.328 | -0.021 | 0.0027 | 1.27E-14 | Yes | Yes |
| rs6948707 | 7 | 1870794 | T | G | 0.419 | 0.024 | 0.0026 | 4.24E-21 | Yes | Yes |
| rs10259715 | 7 | 3329967 | T | A | 0.210 | -0.019 | 0.0032 | 6.42E-09 | No | No |
| rs13237637 | 7 | 3503207 | G | C | 0.485 | -0.024 | 0.0025 | 1.54E-20 | Yes | No |
| rs79631993 | 7 | 69432311 | A | C | 0.216 | -0.017 | 0.0031 | 3.67E-08 | No | Yes |
| rs7809303 | 7 | 69484366 | G | A | 0.325 | -0.021 | 0.0027 | 3.48E-15 | Yes | Yes |
| rs7802996 | 7 | 77771983 | C | T | 0.166 | -0.021 | 0.0034 | 1.06E-09 | Yes | Yes |
| rs1030015 | 7 | 78139581 | G | T | 0.520 | 0.014 | 0.0026 | 2.15E-08 | Yes | Yes |
| rs4727189 | 7 | 88442568 | T | C | 0.344 | 0.015 | 0.0027 | 3.00E-08 | Yes | Yes |
| rs76841737 | 7 | 91281409 | C | G | 0.103 | -0.023 | 0.0042 | 3.26E-08 | Yes | Yes |
| rs11768481 | 7 | 96629103 | C | A | 0.340 | -0.019 | 0.0027 | 5.23E-12 | Yes | Yes |
| rs1799068 | 7 | 97707069 | G | T | 0.379 | 0.017 | 0.0026 | 2.59E-10 | Yes | Yes |
| rs13437771 | 7 | 99071478 | A | G | 0.155 | -0.027 | 0.0035 | 1.39E-14 | Yes | Yes |
| rs11766326 | 7 | 111100585 | T | C | 0.506 | -0.018 | 0.0026 | 1.79E-11 | Yes | Yes |
| rs6968380 | 7 | 114940159 | G | A | 0.681 | -0.023 | 0.0027 | 1.05E-17 | Yes | Yes |
| rs112913817 | 7 | 115077394 | A | G | 0.011 | 0.078 | 0.0120 | 9.28E-11 | Yes | Yes |
| rs10233018 | 7 | 117523709 | A | G | 0.516 | 0.025 | 0.0025 | 4.77E-22 | Yes | Yes |
| rs10953957 | 7 | 121954709 | G | A | 0.386 | 0.014 | 0.0026 | 3.66E-08 | Yes | Yes |
| rs77283305 | 7 | 132593831 | G | A | 0.306 | -0.015 | 0.0028 | 3.91E-08 | Yes | Yes |
| rs10279261 | 7 | 133589846 | G | A | 0.618 | -0.019 | 0.0026 | 6.05E-13 | Yes | Yes |
| rs1561112 | 7 | 133840652 | T | C | 0.413 | -0.015 | 0.0026 | 3.84E-09 | Yes | Yes |
| rs2952251 | 8 | 10143164 | A | G | 0.744 | 0.016 | 0.0030 | 4.24E-08 | Yes | Yes |
| rs4326350 | 8 | 10763655 | C | G | 0.493 | -0.018 | 0.0026 | 5.16E-12 | Yes | No |
| rs11780471 | 8 | 27344719 | G | A | 0.063 | -0.039 | 0.0052 | 1.57E-13 | Yes | Yes |
| rs11783093 | 8 | 27425349 | C | T | 0.158 | -0.047 | 0.0035 | 2.07E-41 | Yes | Yes |
| rs1565735 | 8 | 27426077 | T | A | 0.204 | -0.019 | 0.0032 | 1.33E-09 | Yes | Yes |
| rs7836565 | 8 | 52569449 | C | T | 0.718 | -0.016 | 0.0028 | 4.36E-08 | Yes | Yes |
| rs13261666 | 8 | 59814666 | G | T | 0.517 | -0.020 | 0.0025 | 4.36E-15 | Yes | Yes |
| rs3850736 | 8 | 64912021 | C | G | 0.474 | 0.019 | 0.0026 | 6.43E-14 | Yes | No |
| rs2063976 | 8 | 91096366 | C | T | 0.665 | -0.020 | 0.0027 | 7.45E-14 | Yes | Yes |
| rs6993429 | 8 | 92733282 | C | A | 0.453 | -0.019 | 0.0026 | 9.87E-14 | Yes | Yes |
| rs6986430 | 8 | 93048104 | T | C | 0.222 | -0.024 | 0.0031 | 1.99E-15 | Yes | Yes |
| rs9987376 | 8 | 93190014 | T | G | 0.574 | -0.020 | 0.0026 | 2.01E-15 | Yes | Yes |
| rs290601 | 8 | 115374642 | C | T | 0.274 | 0.016 | 0.0029 | 1.14E-08 | Yes | Yes |
| rs3847244 | 9 | 3025368 | C | T | 0.470 | 0.019 | 0.0026 | 2.60E-13 | Yes | Yes |
| rs11791671 | 9 | 3398679 | C | T | 0.067 | 0.028 | 0.0051 | 4.24E-08 | Yes | Yes |
| rs7024924 | 9 | 8282399 | T | C | 0.174 | 0.019 | 0.0034 | 1.90E-08 | Yes | Yes |
| rs6474609 | 9 | 10981069 | T | A | 0.587 | -0.016 | 0.0026 | 1.71E-09 | Yes | No |
| rs1931431 | 9 | 11161799 | G | C | 0.478 | 0.018 | 0.0026 | 8.56E-13 | Yes | No |
| rs7867822 | 9 | 20676454 | A | G | 0.673 | -0.015 | 0.0027 | 2.76E-08 | Yes | Yes |
| rs10966092 | 9 | 23831658 | T | C | 0.267 | -0.020 | 0.0029 | 1.12E-12 | Yes | Yes |
| rs10969352 | 9 | 29747488 | T | A | 0.500 | 0.014 | 0.0025 | 1.82E-08 | Yes | No |
| rs4877285 | 9 | 81354129 | G | A | 0.668 | -0.018 | 0.0027 | 2.10E-11 | Yes | Yes |
| rs1930371 | 9 | 81444104 | C | T | 0.241 | -0.017 | 0.0030 | 7.09E-09 | Yes | Yes |
| rs2378662 | 9 | 86707289 | G | A | 0.541 | 0.015 | 0.0026 | 2.67E-09 | Yes | Yes |
| rs1927901 | 9 | 120519111 | T | C | 0.553 | -0.014 | 0.0026 | 3.10E-08 | Yes | Yes |
| rs4837631 | 9 | 122061948 | C | T | 0.446 | -0.015 | 0.0026 | 2.03E-09 | Yes | Yes |
| rs1759433 | 9 | 128073097 | G | A | 0.480 | 0.015 | 0.0026 | 1.69E-09 | Yes | Yes |
| rs34553878 | 9 | 134334588 | A | G | 0.111 | 0.025 | 0.0041 | 1.17E-09 | Yes | Yes |
| rs7026534 | 9 | 134907263 | T | G | 0.704 | -0.017 | 0.0028 | 2.68E-09 | Yes | Yes |
| rs10858334 | 9 | 137989785 | C | G | 0.140 | 0.023 | 0.0038 | 1.18E-09 | Yes | Yes |
| rs10905461 | 10 | 8803551 | T | C | 0.748 | -0.016 | 0.0029 | 2.36E-08 | Yes | Yes |
| rs7920501 | 10 | 10043159 | T | A | 0.465 | -0.016 | 0.0026 | 1.25E-09 | Yes | No |
| rs1291821 | 10 | 11133823 | A | G | 0.534 | 0.014 | 0.0026 | 1.39E-08 | Yes | Yes |
| rs11258417 | 10 | 13533053 | C | T | 0.391 | -0.015 | 0.0026 | 2.71E-08 | Yes | Yes |
| rs7072776 | 10 | 22032942 | A | G | 0.712 | -0.022 | 0.0028 | 5.66E-15 | Yes | Yes |
| rs2796793 | 10 | 36634124 | G | A | 0.452 | 0.014 | 0.0026 | 1.55E-08 | Yes | Yes |
| rs1733760 | 10 | 56698174 | T | C | 0.510 | 0.015 | 0.0025 | 6.70E-09 | Yes | Yes |
| rs7921378 | 10 | 63674885 | G | C | 0.482 | -0.023 | 0.0025 | 6.10E-20 | Yes | No |
| rs7901883 | 10 | 103186838 | G | A | 0.230 | -0.019 | 0.0030 | 1.98E-10 | Yes | Yes |
| rs11594623 | 10 | 103960351 | T | C | 0.234 | 0.027 | 0.0030 | 7.45E-20 | Yes | Yes |
| rs11191269 | 10 | 104120522 | C | G | 0.193 | 0.018 | 0.0032 | 4.61E-08 | Yes | Yes |
| rs28408682 | 10 | 104403310 | A | G | 0.600 | 0.017 | 0.0026 | 1.41E-10 | Yes | Yes |
| rs12244388 | 10 | 104640052 | G | A | 0.350 | 0.026 | 0.0027 | 4.31E-22 | Yes | Yes |
| rs111842178 | 10 | 104852121 | A | G | 0.231 | 0.022 | 0.0032 | 2.24E-12 | No | No |
| rs34970111 | 10 | 106078937 | C | T | 0.458 | -0.015 | 0.0026 | 1.28E-08 | No | Yes |
| rs9787523 | 10 | 106460460 | T | C | 0.418 | -0.016 | 0.0026 | 1.42E-09 | Yes | Yes |
| rs11192347 | 10 | 106929313 | G | A | 0.104 | -0.026 | 0.0043 | 6.15E-10 | Yes | Yes |
| rs10885480 | 10 | 115378364 | T | C | 0.284 | -0.019 | 0.0028 | 3.83E-11 | Yes | Yes |
| rs4752018 | 10 | 118678712 | C | A | 0.231 | 0.019 | 0.0030 | 4.42E-10 | Yes | Yes |
| rs9423279 | 10 | 125680419 | C | G | 0.645 | -0.019 | 0.0027 | 3.06E-12 | No | Yes |
| rs6265 | 11 | 27679916 | C | T | 0.188 | -0.029 | 0.0033 | 2.81E-19 | Yes | Yes |
| rs4275621 | 11 | 28652996 | A | G | 0.382 | -0.021 | 0.0026 | 3.76E-16 | Yes | Yes |
| rs62618693 | 11 | 32956492 | C | T | 0.043 | -0.035 | 0.0063 | 2.09E-08 | Yes | Yes |
| rs2939756 | 11 | 41436297 | G | A | 0.480 | -0.016 | 0.0026 | 7.45E-10 | Yes | Yes |
| rs1381775 | 11 | 42442826 | T | C | 0.712 | -0.016 | 0.0028 | 2.79E-08 | Yes | Yes |
| rs2959084 | 11 | 46078656 | G | A | 0.705 | 0.017 | 0.0028 | 9.82E-10 | Yes | Yes |
| rs3740977 | 11 | 46393574 | T | C | 0.167 | 0.019 | 0.0034 | 1.17E-08 | Yes | Yes |
| rs61886926 | 11 | 64133552 | C | T | 0.384 | -0.018 | 0.0026 | 7.30E-12 | Yes | Yes |
| rs61884449 | 11 | 64485193 | C | T | 0.149 | 0.020 | 0.0036 | 2.32E-08 | Yes | Yes |
| rs644740 | 11 | 65561468 | C | T | 0.457 | -0.014 | 0.0026 | 3.67E-08 | Yes | Yes |
| rs7943721 | 11 | 73309393 | G | A | 0.829 | -0.021 | 0.0034 | 3.58E-10 | Yes | Yes |
| rs7929518 | 11 | 85980958 | A | G | 0.773 | 0.019 | 0.0030 | 2.55E-10 | Yes | Yes |
| rs586699 | 11 | 92289734 | G | A | 0.543 | -0.015 | 0.0026 | 7.29E-09 | Yes | Yes |
| rs76460663 | 11 | 111979741 | C | G | 0.041 | -0.042 | 0.0064 | 4.15E-11 | Yes | Yes |
| rs2155646 | 11 | 112912811 | T | C | 0.400 | 0.038 | 0.0026 | 9.44E-48 | Yes | Yes |
| rs78239456 | 11 | 112984491 | A | T | 0.377 | -0.018 | 0.0027 | 9.37E-12 | No | No |
| rs1713676 | 11 | 113660576 | A | G | 0.523 | -0.017 | 0.0026 | 5.38E-11 | Yes | Yes |
| rs238896 | 11 | 113994505 | G | A | 0.490 | -0.017 | 0.0025 | 3.65E-11 | Yes | Yes |
| rs540860 | 11 | 121530888 | A | G | 0.543 | 0.018 | 0.0026 | 5.75E-12 | Yes | Yes |
| rs1944689 | 11 | 121634334 | G | T | 0.786 | 0.018 | 0.0031 | 1.27E-08 | Yes | Yes |
| rs1834306 | 11 | 122023187 | A | G | 0.579 | -0.014 | 0.0026 | 1.96E-08 | Yes | Yes |
| rs1106363 | 11 | 131966264 | C | T | 0.345 | 0.017 | 0.0027 | 9.20E-11 | Yes | Yes |
| rs2010921 | 11 | 132098205 | G | A | 0.311 | 0.017 | 0.0028 | 2.47E-10 | Yes | Yes |
| rs11057005 | 12 | 16748721 | A | G | 0.441 | -0.016 | 0.0026 | 9.12E-10 | Yes | Yes |
| rs13906 | 12 | 49952394 | C | T | 0.109 | -0.025 | 0.0041 | 1.98E-09 | Yes | Yes |
| rs4759229 | 12 | 56474480 | A | G | 0.656 | 0.016 | 0.0027 | 6.53E-09 | Yes | Yes |
| rs7969559 | 12 | 69655167 | A | G | 0.713 | -0.017 | 0.0028 | 1.53E-09 | Yes | Yes |
| rs7134009 | 12 | 75263193 | T | C | 0.287 | -0.016 | 0.0029 | 4.30E-08 | Yes | Yes |
| rs77215829 | 12 | 112618346 | A | C | 0.131 | -0.024 | 0.0038 | 2.02E-10 | Yes | Yes |
| rs1109480 | 12 | 121083279 | G | A | 0.384 | -0.017 | 0.0026 | 1.84E-10 | Yes | Yes |
| rs11611651 | 12 | 133380790 | G | A | 0.087 | 0.027 | 0.0045 | 2.05E-09 | Yes | Yes |
| rs17197663 | 13 | 38172867 | G | A | 0.125 | -0.022 | 0.0039 | 2.06E-08 | Yes | Yes |
| rs4264267 | 13 | 38359676 | C | T | 0.527 | 0.015 | 0.0026 | 6.82E-09 | Yes | Yes |
| rs61959481 | 13 | 55834929 | G | A | 0.210 | -0.020 | 0.0031 | 7.95E-11 | Yes | Yes |
| rs3098272 | 13 | 55931424 | A | C | 0.799 | -0.018 | 0.0032 | 2.08E-08 | Yes | Yes |
| rs9538162 | 13 | 59265043 | T | C | 0.416 | 0.017 | 0.0026 | 1.76E-11 | Yes | Yes |
| rs1413119 | 13 | 59339281 | C | T | 0.396 | -0.015 | 0.0026 | 4.77E-09 | Yes | Yes |
| rs56367474 | 13 | 59454139 | C | T | 0.304 | -0.017 | 0.0028 | 4.20E-10 | Yes | Yes |
| rs55786907 | 13 | 59871584 | A | G | 0.162 | 0.019 | 0.0035 | 1.84E-08 | Yes | Yes |
| rs4886207 | 13 | 60705792 | T | C | 0.637 | -0.016 | 0.0026 | 8.78E-10 | Yes | Yes |
| rs9540731 | 13 | 66949370 | C | T | 0.509 | -0.018 | 0.0025 | 3.42E-12 | Yes | Yes |
| rs9545155 | 13 | 80191873 | T | C | 0.478 | -0.016 | 0.0026 | 3.04E-10 | Yes | Yes |
| rs1772572 | 13 | 81191176 | C | A | 0.324 | -0.017 | 0.0027 | 5.62E-10 | Yes | Yes |
| rs75674569 | 13 | 96823724 | G | A | 0.100 | -0.025 | 0.0043 | 2.58E-09 | Yes | Yes |
| rs7333559 | 13 | 100546450 | G | A | 0.783 | -0.023 | 0.0031 | 5.94E-14 | Yes | Yes |
| rs1108130 | 13 | 100648356 | T | A | 0.212 | 0.024 | 0.0031 | 1.57E-14 | Yes | Yes |
| rs12855717 | 13 | 101252635 | C | T | 0.538 | 0.016 | 0.0026 | 1.22E-09 | Yes | Yes |
| rs12878369 | 14 | 28346502 | C | A | 0.415 | 0.017 | 0.0026 | 1.60E-11 | Yes | Yes |
| rs2145451 | 14 | 29316842 | T | C | 0.193 | -0.020 | 0.0032 | 5.44E-10 | No | Yes |
| rs9323328 | 14 | 58653514 | A | G | 0.537 | -0.014 | 0.0026 | 2.55E-08 | Yes | Yes |
| rs1811739 | 14 | 77529375 | G | A | 0.248 | 0.018 | 0.0030 | 5.97E-10 | Yes | Yes |
| rs8005334 | 14 | 79563654 | T | G | 0.360 | 0.017 | 0.0027 | 3.44E-10 | Yes | Yes |
| rs34940743 | 14 | 80102233 | A | G | 0.346 | 0.016 | 0.0027 | 2.80E-09 | No | Yes |
| rs2925128 | 14 | 98362355 | C | T | 0.385 | 0.017 | 0.0027 | 3.67E-10 | Yes | Yes |
| rs1381287 | 14 | 98597552 | C | T | 0.467 | 0.018 | 0.0026 | 1.81E-12 | Yes | Yes |
| rs55913542 | 14 | 99693843 | G | T | 0.175 | 0.019 | 0.0034 | 3.25E-08 | Yes | Yes |
| rs1435672 | 15 | 36399479 | T | C | 0.560 | 0.014 | 0.0026 | 3.82E-08 | Yes | Yes |
| rs281296 | 15 | 47685010 | G | A | 0.357 | 0.025 | 0.0027 | 1.59E-20 | Yes | Yes |
| rs1435741 | 15 | 47935843 | G | A | 0.433 | 0.018 | 0.0026 | 1.09E-12 | Yes | Yes |
| rs56902655 | 15 | 63898709 | T | G | 0.136 | -0.022 | 0.0037 | 4.09E-09 | Yes | Yes |
| rs2289791 | 15 | 67476952 | G | T | 0.247 | -0.018 | 0.0030 | 2.01E-09 | Yes | Yes |
| rs60833441 | 15 | 74048768 | A | G | 0.461 | -0.014 | 0.0026 | 2.28E-08 | Yes | Yes |
| rs62007780 | 15 | 78025464 | G | T | 0.416 | -0.016 | 0.0026 | 7.48E-10 | Yes | Yes |
| rs12442563 | 15 | 83893243 | G | T | 0.223 | -0.023 | 0.0031 | 3.13E-14 | No | Yes |
| rs4310804 | 15 | 96858409 | C | G | 0.247 | -0.018 | 0.0030 | 7.55E-10 | Yes | Yes |
| rs8027457 | 15 | 99204101 | T | C | 0.511 | 0.015 | 0.0025 | 1.88E-09 | Yes | Yes |
| rs1139897 | 16 | 720986 | G | A | 0.230 | -0.024 | 0.0030 | 1.77E-15 | Yes | Yes |
| rs11076962 | 16 | 5811367 | T | C | 0.279 | 0.018 | 0.0028 | 1.20E-10 | Yes | Yes |
| rs7192140 | 16 | 10173748 | T | C | 0.498 | -0.017 | 0.0025 | 3.40E-11 | Yes | Yes |
| rs9922607 | 16 | 17570220 | C | T | 0.200 | -0.022 | 0.0032 | 3.42E-12 | Yes | Yes |
| rs9941217 | 16 | 18050926 | C | G | 0.352 | -0.019 | 0.0027 | 3.50E-12 | Yes | Yes |
| rs7188873 | 16 | 24727064 | A | G | 0.613 | 0.020 | 0.0026 | 8.46E-15 | Yes | Yes |
| rs6497840 | 16 | 25351633 | G | A | 0.707 | 0.023 | 0.0029 | 2.01E-15 | Yes | Yes |
| rs4785187 | 16 | 49766772 | G | A | 0.223 | 0.020 | 0.0031 | 6.55E-11 | Yes | Yes |
| rs8050598 | 16 | 49891964 | C | T | 0.254 | 0.019 | 0.0029 | 1.76E-10 | Yes | Yes |
| rs12918191 | 16 | 50945156 | A | G | 0.243 | -0.020 | 0.0030 | 3.14E-11 | Yes | Yes |
| rs9302604 | 16 | 69576894 | A | G | 0.435 | 0.019 | 0.0026 | 3.29E-13 | Yes | Yes |
| rs9936784 | 16 | 72230694 | T | G | 0.534 | 0.014 | 0.0026 | 4.33E-08 | Yes | Yes |
| rs62052916 | 16 | 72574550 | A | T | 0.070 | -0.032 | 0.0050 | 1.62E-10 | Yes | Yes |
| rs4788676 | 16 | 72950468 | T | C | 0.229 | -0.018 | 0.0030 | 4.92E-09 | Yes | Yes |
| rs61537885 | 16 | 75620118 | T | C | 0.037 | -0.040 | 0.0069 | 8.06E-09 | No | No |
| rs117657830 | 16 | 75766873 | A | G | 0.042 | -0.038 | 0.0064 | 3.18E-09 | Yes | Yes |
| rs1050847 | 16 | 87443734 | C | T | 0.559 | -0.015 | 0.0026 | 7.37E-09 | Yes | Yes |
| rs11642231 | 16 | 89608702 | G | A | 0.369 | -0.016 | 0.0026 | 3.44E-09 | Yes | Yes |
| rs4790874 | 17 | 1995177 | C | T | 0.532 | 0.017 | 0.0026 | 8.43E-12 | Yes | Yes |
| rs11078713 | 17 | 7795972 | A | G | 0.419 | -0.015 | 0.0026 | 1.59E-08 | Yes | Yes |
| rs28441558 | 17 | 7803118 | T | C | 0.056 | -0.036 | 0.0055 | 1.24E-10 | Yes | Yes |
| rs11651955 | 17 | 16235462 | G | A | 0.499 | -0.014 | 0.0025 | 3.74E-08 | Yes | Yes |
| rs67777803 | 17 | 27323322 | G | T | 0.172 | -0.025 | 0.0034 | 3.18E-13 | Yes | Yes |
| rs2344976 | 17 | 30685935 | T | C | 0.612 | -0.015 | 0.0026 | 7.98E-09 | Yes | Yes |
| rs3764351 | 17 | 37824339 | G | A | 0.657 | -0.015 | 0.0027 | 3.89E-08 | Yes | Yes |
| rs72836318 | 17 | 44121579 | T | C | 0.246 | -0.017 | 0.0030 | 7.00E-09 | No | Yes |
| rs17692129 | 17 | 44793283 | C | T | 0.331 | 0.020 | 0.0027 | 4.57E-13 | No | Yes |
| rs75919030 | 17 | 50193197 | T | C | 0.267 | -0.021 | 0.0029 | 3.35E-13 | Yes | Yes |
| rs2938134 | 17 | 50243397 | C | A | 0.673 | -0.018 | 0.0028 | 3.14E-10 | Yes | Yes |
| rs2587507 | 17 | 77790135 | T | C | 0.502 | -0.015 | 0.0025 | 8.69E-09 | Yes | Yes |
| rs34342129 | 18 | 5872472 | T | C | 0.509 | -0.014 | 0.0025 | 2.13E-08 | Yes | Yes |
| rs4476253 | 18 | 25253297 | G | A | 0.240 | -0.018 | 0.0030 | 5.78E-10 | Yes | Yes |
| rs7505855 | 18 | 31696075 | C | T | 0.586 | -0.017 | 0.0026 | 5.31E-11 | Yes | Yes |
| rs8096225 | 18 | 36921851 | A | C | 0.703 | 0.016 | 0.0028 | 2.63E-08 | Yes | Yes |
| rs67050670 | 18 | 39297254 | A | G | 0.229 | -0.020 | 0.0030 | 2.34E-11 | Yes | Yes |
| rs2359180 | 18 | 41314171 | A | G | 0.369 | -0.014 | 0.0026 | 4.98E-08 | No | Yes |
| rs72898831 | 18 | 42658643 | A | G | 0.155 | -0.024 | 0.0035 | 4.14E-12 | Yes | Yes |
| rs8083764 | 18 | 49874515 | G | T | 0.306 | -0.016 | 0.0028 | 7.97E-09 | Yes | Yes |
| rs1373178 | 18 | 49967811 | T | G | 0.588 | -0.020 | 0.0026 | 4.16E-15 | Yes | Yes |
| rs62098013 | 18 | 50863861 | G | A | 0.365 | 0.018 | 0.0026 | 2.24E-11 | Yes | Yes |
| rs72938304 | 18 | 53661743 | G | A | 0.113 | -0.027 | 0.0040 | 1.36E-11 | Yes | Yes |
| rs11872397 | 18 | 72535282 | G | A | 0.253 | -0.017 | 0.0029 | 5.20E-09 | Yes | Yes |
| rs71367544 | 18 | 77574374 | C | T | 0.203 | 0.021 | 0.0032 | 8.54E-11 | Yes | Yes |
| rs76608582 | 19 | 4474725 | C | A | 0.049 | -0.035 | 0.0059 | 4.88E-09 | Yes | Yes |
| rs10853981 | 19 | 4965064 | G | A | 0.330 | 0.015 | 0.0027 | 4.88E-08 | Yes | Yes |
| rs113230003 | 19 | 18460956 | G | A | 0.255 | -0.019 | 0.0029 | 1.05E-10 | Yes | Yes |
| rs8103660 | 19 | 18566395 | T | C | 0.354 | 0.016 | 0.0027 | 3.03E-09 | Yes | Yes |
| rs117734003 | 19 | 51129745 | G | C | 0.067 | 0.030 | 0.0051 | 2.57E-09 | Yes | Yes |
| rs1126757 | 19 | 55879872 | C | T | 0.473 | 0.014 | 0.0026 | 2.92E-08 | Yes | Yes |
| rs6050446 | 20 | 25195509 | A | G | 0.971 | 0.054 | 0.0076 | 8.80E-13 | Yes | Yes |
| rs6058782 | 20 | 29946968 | C | T | 0.908 | 0.030 | 0.0044 | 1.78E-11 | Yes | Yes |
| rs1555445 | 20 | 31175258 | A | T | 0.318 | 0.019 | 0.0027 | 7.75E-12 | Yes | Yes |
| rs6073075 | 20 | 42015801 | T | A | 0.824 | -0.019 | 0.0034 | 2.44E-08 | Yes | Yes |
| rs910912 | 20 | 54462393 | T | C | 0.739 | -0.017 | 0.0029 | 7.82E-09 | Yes | Yes |
| rs6011779 | 20 | 61984317 | C | T | 0.806 | -0.019 | 0.0032 | 2.83E-09 | Yes | Yes |
| rs3810496 | 20 | 62406886 | T | C | 0.619 | 0.016 | 0.0026 | 1.54E-09 | Yes | Yes |
| rs4818005 | 21 | 40588819 | G | A | 0.581 | -0.020 | 0.0026 | 1.09E-14 | Yes | Yes |
| rs139896 | 22 | 38397797 | T | C | 0.648 | 0.015 | 0.0027 | 7.14E-09 | Yes | Yes |
| rs4822102 | 22 | 42698430 | C | T | 0.618 | -0.017 | 0.0026 | 2.78E-10 | Yes | Yes |
| rs9627272 | 22 | 46442288 | G | C | 0.407 | -0.015 | 0.0026 | 2.42E-09 | No | Yes |

# **Supplementary Table S2.** Genetic instruments for smoking intensity (cigarettes smoked per day)

| **RSID** | **Chrom.** | **Position** | **Reference**  **Allele** | **Effect**  **Allele** | **Effect**  **Allele**  **Freq.** | **Beta** | **SE** | **p-value** | **One-**  **sample**  **MR** | **Two-**  **sample**  **MR** |
| --- | --- | --- | --- | --- | --- | --- | --- | --- | --- | --- |
| rs11264100 | 1 | 35591626 | A | G | 0.876 | -0.022 | 0.0037 | 2.22E-09 | Yes | Yes |
| rs2072659 | 1 | 154548521 | C | G | 0.099 | -0.030 | 0.0041 | 2.51E-13 | Yes | Yes |
| rs34973462 | 1 | 175993820 | C | T | 0.334 | 0.015 | 0.0026 | 5.85E-09 | Yes | Yes |
| rs7599488 | 2 | 60718347 | C | T | 0.437 | 0.014 | 0.0025 | 8.95E-09 | Yes | Yes |
| rs78408772 | 2 | 62710608 | C | T | 0.102 | -0.022 | 0.0040 | 4.51E-08 | Yes | Yes |
| rs10204824 | 2 | 148372720 | A | G | 0.639 | -0.018 | 0.0025 | 1.35E-12 | Yes | Yes |
| rs2084533 | 3 | 16872929 | C | T | 0.321 | 0.016 | 0.0026 | 6.53E-10 | Yes | Yes |
| rs7431710 | 3 | 48935583 | G | A | 0.654 | -0.018 | 0.0026 | 1.04E-12 | Yes | Yes |
| rs2236951 | 3 | 50421081 | T | C | 0.200 | -0.017 | 0.0030 | 1.59E-08 | Yes | Yes |
| rs699165 | 3 | 136224697 | A | G | 0.745 | 0.016 | 0.0028 | 8.09E-09 | Yes | Yes |
| rs28813180 | 3 | 158083918 | G | A | 0.498 | -0.015 | 0.0024 | 1.95E-10 | Yes | Yes |
| rs1024323 | 4 | 3006043 | C | T | 0.382 | -0.014 | 0.0025 | 8.66E-09 | Yes | Yes |
| rs11940255 | 4 | 67086288 | G | A | 0.717 | -0.017 | 0.0027 | 2.20E-10 | Yes | Yes |
| rs10454798 | 4 | 67980830 | G | T | 0.253 | 0.016 | 0.0028 | 1.53E-08 | Yes | Yes |
| rs7766641 | 6 | 26184102 | G | A | 0.272 | -0.017 | 0.0027 | 2.91E-10 | Yes | Yes |
| rs215600 | 7 | 32333642 | G | A | 0.645 | -0.024 | 0.0025 | 4.02E-21 | Yes | Yes |
| rs62447179 | 7 | 50339609 | G | A | 0.298 | -0.015 | 0.0027 | 9.68E-09 | Yes | Yes |
| rs2741351 | 8 | 27418040 | A | C | 0.826 | 0.018 | 0.0032 | 8.80E-09 | Yes | Yes |
| rs73229090 | 8 | 27442127 | C | A | 0.112 | 0.026 | 0.0039 | 1.14E-11 | Yes | Yes |
| rs13253502 | 8 | 42442018 | G | A | 0.407 | -0.014 | 0.0025 | 2.31E-08 | Yes | Yes |
| rs4236926 | 8 | 42578059 | T | G | 0.766 | 0.034 | 0.0029 | 7.66E-33 | Yes | Yes |
| rs790564 | 8 | 64604218 | A | C | 0.729 | -0.018 | 0.0027 | 1.24E-10 | Yes | Yes |
| rs75596189 | 9 | 136468701 | C | T | 0.112 | 0.036 | 0.0039 | 1.84E-20 | Yes | Yes |
| rs3025383 | 9 | 136502369 | T | C | 0.187 | -0.031 | 0.0031 | 9.78E-24 | Yes | Yes |
| rs7951365 | 11 | 16377044 | T | C | 0.310 | 0.018 | 0.0026 | 1.53E-11 | Yes | Yes |
| rs10742683 | 11 | 43667625 | G | A | 0.415 | -0.013 | 0.0025 | 4.83E-08 | Yes | Yes |
| rs113001570 | 11 | 46737412 | A | T | 0.067 | 0.030 | 0.0049 | 1.04E-09 | Yes | Yes |
| rs7125588 | 11 | 113436072 | A | G | 0.429 | -0.017 | 0.0025 | 6.50E-12 | Yes | Yes |
| rs11846838 | 14 | 104184737 | G | A | 0.327 | 0.015 | 0.0026 | 5.03E-09 | Yes | Yes |
| rs1115019 | 15 | 57141231 | T | C | 0.790 | -0.018 | 0.0030 | 2.27E-09 | Yes | Yes |
| rs632811 | 15 | 59155050 | A | G | 0.330 | -0.018 | 0.0028 | 1.67E-10 | Yes | Yes |
| rs4886550 | 15 | 78243579 | A | G | 0.288 | -0.020 | 0.0034 | 4.58E-09 | No | No |
| rs12438181 | 15 | 78812098 | G | A | 0.218 | -0.019 | 0.0030 | 4.97E-10 | Yes | Yes |
| rs10519203 | 15 | 78814046 | G | A | 0.655 | -0.094 | 0.0026 | 3.12E-286 | Yes | Yes |
| rs28438420 | 15 | 78836288 | A | T | 0.554 | 0.018 | 0.0025 | 1.25E-12 | Yes | No |
| rs72740955 | 15 | 78849779 | C | T | 0.337 | 0.032 | 0.0026 | 2.42E-34 | Yes | Yes |
| rs146009840 | 15 | 78906177 | A | T | 0.335 | 0.022 | 0.0026 | 2.00E-17 | Yes | Yes |
| rs28681284 | 15 | 78908565 | C | T | 0.210 | -0.049 | 0.0030 | 2.10E-58 | Yes | Yes |
| rs8040868 | 15 | 78911181 | T | C | 0.400 | 0.016 | 0.0025 | 1.79E-10 | Yes | Yes |
| rs3743063 | 15 | 79065171 | A | C | 0.562 | -0.017 | 0.0025 | 1.53E-11 | Yes | Yes |
| rs182317 | 15 | 89943601 | G | T | 0.355 | -0.016 | 0.0026 | 1.31E-09 | Yes | Yes |
| rs1592485 | 16 | 52093549 | C | A | 0.611 | -0.016 | 0.0025 | 1.11E-10 | Yes | Yes |
| rs12924872 | 16 | 69552215 | C | T | 0.463 | -0.013 | 0.0024 | 4.39E-08 | Yes | Yes |
| rs258321 | 16 | 89756473 | A | G | 0.429 | 0.016 | 0.0025 | 1.53E-10 | Yes | Yes |
| rs4144686 | 18 | 53251725 | G | A | 0.167 | -0.019 | 0.0033 | 1.35E-08 | Yes | Yes |
| rs4485470 | 18 | 62125063 | G | A | 0.592 | -0.015 | 0.0025 | 7.05E-10 | Yes | Yes |
| rs59208569 | 19 | 4044424 | G | C | 0.829 | 0.020 | 0.0032 | 2.45E-10 | Yes | Yes |
| rs143200968 | 19 | 41338847 | G | C | 0.025 | -0.086 | 0.0079 | 6.97E-28 | Yes | Yes |
| rs56113850 | 19 | 41353107 | T | C | 0.555 | 0.052 | 0.0025 | 4.01E-99 | No | Yes |
| rs8192726 | 19 | 41354496 | C | A | 0.068 | -0.039 | 0.0049 | 8.35E-16 | Yes | Yes |
| rs117824460 | 19 | 41371480 | A | G | 0.026 | -0.095 | 0.0077 | 7.66E-35 | Yes | Yes |
| rs6078373 | 20 | 11863500 | G | A | 0.402 | 0.016 | 0.0025 | 9.40E-11 | Yes | Yes |
| rs1737894 | 20 | 31054702 | C | G | 0.408 | 0.017 | 0.0025 | 9.90E-12 | Yes | No |
| rs2273500 | 20 | 61986949 | T | C | 0.147 | 0.036 | 0.0034 | 3.49E-26 | Yes | Yes |
| rs7281463 | 21 | 40520783 | A | C | 0.413 | 0.014 | 0.0025 | 3.15E-08 | Yes | Yes |

# **Supplementary Table S3.** Genetic instruments for smoking cessation

| **RSID** | **Chrom.** | **Position** | **Reference**  **Allele** | **Effect**  **Allele** | **Effect**  **Allele**  **Freq.** | **Beta** | **SE** | **p-value** | **One-**  **sample**  **MR** | **Two-**  **sample**  **MR** |
| --- | --- | --- | --- | --- | --- | --- | --- | --- | --- | --- |
| rs112187834 | 2 | 23953454 | T | A | 0.140 | 0.033 | 0.0056 | 2.81E-09 | Yes | Yes |
| rs7617480 | 3 | 49210732 | A | C | 0.773 | -0.033 | 0.0047 | 1.68E-12 | Yes | Yes |
| rs12203592 | 6 | 396321 | C | T | 0.176 | -0.029 | 0.0051 | 1.21E-08 | Yes | Yes |
| rs707968 | 6 | 35058117 | A | G | 0.681 | 0.023 | 0.0042 | 2.76E-08 | Yes | Yes |
| rs7778443 | 7 | 32314690 | T | C | 0.618 | -0.023 | 0.0040 | 1.04E-08 | Yes | Yes |
| rs1565735 | 8 | 27426077 | T | A | 0.199 | -0.035 | 0.0049 | 1.54E-12 | Yes | Yes |
| rs60749569 | 8 | 42602668 | A | T | 0.080 | -0.040 | 0.0072 | 2.68E-08 | Yes | Yes |
| rs12378015 | 9 | 127917257 | G | A | 0.300 | -0.028 | 0.0043 | 8.31E-11 | Yes | Yes |
| rs9409844 | 9 | 136461851 | G | A | 0.045 | -0.059 | 0.0094 | 4.37E-10 | Yes | Yes |
| rs3025327 | 9 | 136467344 | G | C | 0.107 | 0.079 | 0.0063 | 1.19E-35 | Yes | Yes |
| rs10821523 | 9 | 136473572 | A | C | 0.536 | 0.026 | 0.0039 | 2.28E-11 | No | Yes |
| rs1611124 | 9 | 136509275 | G | T | 0.068 | -0.045 | 0.0078 | 5.26E-09 | Yes | Yes |
| rs7109376 | 11 | 16372431 | T | A | 0.279 | 0.028 | 0.0044 | 1.14E-10 | Yes | Yes |
| rs591143 | 15 | 47647755 | C | T | 0.592 | -0.024 | 0.0040 | 1.14E-09 | Yes | Yes |
| rs3866543 | 15 | 76629609 | T | G | 0.523 | 0.022 | 0.0039 | 1.35E-08 | Yes | Yes |
| rs518425 | 15 | 78883813 | A | G | 0.285 | -0.031 | 0.0043 | 1.72E-12 | Yes | Yes |
| rs145580088 | 19 | 41342842 | A | G | 0.024 | 0.091 | 0.0127 | 9.48E-13 | Yes | Yes |
| rs56113850 | 19 | 41353107 | T | C | 0.567 | -0.058 | 0.0039 | 1.61E-48 | No | Yes |
| rs117824460 | 19 | 41371480 | A | G | 0.027 | 0.086 | 0.0121 | 1.09E-12 | Yes | Yes |
| rs59586387 | 19 | 41375030 | C | G | 0.068 | 0.051 | 0.0078 | 3.37E-11 | Yes | Yes |
| rs6011779 | 20 | 61984317 | C | T | 0.806 | -0.050 | 0.0050 | 9.89E-24 | Yes | Yes |
| rs4809543 | 20 | 61986950 | G | A | 0.076 | 0.044 | 0.0074 | 2.40E-09 | Yes | Yes |
| rs6089904 | 20 | 62018289 | A | T | 0.047 | -0.064 | 0.0093 | 4.01E-12 | Yes | Yes |
| rs9607805 | 22 | 41854446 | C | T | 0.725 | 0.030 | 0.0044 | 1.37E-11 | Yes | Yes |

# **Supplementary Table S4.** Sex-stratified description of the population

|  | Women  (n = 47,506) | Men  (n = 28,299) |
| --- | --- | --- |
| Age (mean ± SD) | 46.1 ± 4.9 | 48.3 ± 5.4 |
| Education level: |  |  |
| Unfinished high school (*n*, %) | 1,358 (2.86%) | 1,030 (3.7%) |
| High school (*n*, %) | 11,080 (23.3%) | 8,854 (31.4%) |
| College, ≤ 4 years (*n*, %) | 19,951 (42.0%) | 9,489 (33.6%) |
| College, >4 years (*n*, %) | 15,117 (31.8%) | 8,856 (31.4%) |
| Work changes due to COVID-19: |  |  |
| No change (*n*, %) | 35,765 (75.3%) | 19,101 (67.7%) |
| Change to home office (*n*, %) | 4,956 (10.4%) | 3,623 (12.8%) |
| Lost job/sick leave (*n*, %) | 770 (1.6%) | 535 (1.9%) |
| No information (*n*, %) | 6,015 (12.7%) | 4,970 (17.6%) |
| Body mass index: |  |  |
| <25.0 kg/m^2^ (*n*, %) | 25,655 (54.6%) | 9,706 (35.0%) |
| 25.0-29.9 kg/m^2^ (*n*, %) | 13,975 (29.7%) | 13,499 (48.6%) |
| ≥30.0 kg/m^2^ (*n*, %) | 7,359 (15.7%) | 4,544 (16.4%) |
| Number of cohabitants (mean ± SD) | 3.15 ± 0.97 | 3.21 ± 1.01 |
| Region of residence: |  |  |
| Oslo metropolitan area/Viken (*n*, %) | 16,262 (34.2%) | 10,595 (37.5%) |
| Southern Norway (*n*, %) | 8,856 (18.6%) | 5,558 (19.7%) |
| Western Norway (*n*, %) | 7,193 (15.1%) | 3,569 (12.6%) |
| Middle Norway (*n*, %) | 11,787 (24.8%) | 6,436 (22.8%) |
| Northern Norway (*n*, %) | 2,452 (5.2%) | 1,308 (4.6%) |
| Not specified (*n*, %) | 956 (2.0%) | 763 (2.7%) |

# **Supplementary Table S5**. Comparison of MoBa participants included and not included in our analyses

|  | Women | | Men | |
| --- | --- | --- | --- | --- |
|  | Not included | Included | Not included | Included |
| Year of birth, median (1^st^-3^rd^ quartile) | 1975 (1971-1979) | 1974 (1971-1977) | 1973 (1969-1976) | 1972 (1969-1975) |
| Education level: |  |  |  |  |
| Unfinished high school (*n*, %) | 3470 (9.04%) | 2100 (4.5%) | 4132 (10.3%) | 1344 (5.0%) |
| High school (*n*, %) | 12193 (31.8%) | 11686 (25.2%) | 16951 (42.1%) | 8908 (33.1%) |
| College, ≤ 4 years (*n*, %) | 14064 (36.6%) | 20222 (43.6%) | 10278 (25.5%) | 8582 (31.9%) |
| College, >4 years (*n*, %) | 8653 (22.5%) | 12402 (26.7%) | 8896 (22.1%) | 8076 (30.0%) |
| Body mass index: |  |  |  |  |
| <25.0 kg/m^2^ (*n*, %) | 25588 (68.4%) | 30905 (68.0%) | 17592 (44.1%) | 11695 (44.0%) |
| 25.0-29.9 kg/m^2^ (*n*, %) | 7941 (21.2%) | 10211 (22.5%) | 18048 (45.3%) | 12201 (45.9%) |
| ≥30.0 kg/m^2^ (*n*, %) | 3892 (10.4%) | 4338 (9.54%) | 4229 (10.6%) | 2698 (10.1%) |

# **Supplementary Table S6**. Robustness of genetic instruments

| Binary exposures | | | | | | | |
| --- | --- | --- | --- | --- | --- | --- | --- |
|  |  | Number of SNPs in GRS^a^ | GRS ^b^, mean ± SD | OR per +1 SD in GRS | *p*-value | Area under the ROC curve | Pseudo-*R^2^* |
| Having ever smoked | All participants | 351 | 365 ± 11.8 | 1.276, 95% CI 1.257 to 1.295 | <0.001 | 0.57 | 1.85 % |
|  | Women | 351 | 365 ± 11.8 | 1.295, 95% CI 1.269 to 1.321 | <0.001 | 0.57 | 1.95 % |
|  | Men | 351 | 365 ± 11.8 | 1.287, 95% CI 1.257 to 1.318 | <0.001 | 0.57 | 2.06 % |
| Smoking cessation  (current + former smokers) | All participants | 22 | 18.7 ± 2.18 | 1.022, 95% CI 1.007 to 1.038 | 0.005 | 0.51 | 0.015 % |
|  | Women | 22 | 18.7 ± 2.18 | 1.032, 95% CI 1.009 to 1.054 | 0.005 | 0.51 | 0.026 % |
|  | Men | 22 | 18.7 ± 2.17 | 1.015, 95% CI 0.992 to 1.040 | 0.202 | 0.50 | 0.008 % |
| Continuous exposures | | | | | | | |
|  |  | Number of SNPs in GRS | GRS ^b^, mean ± SD | Change per +1 SD in GRS | *p*-value | *F*-statistic | *R^2^* |
| Smoking intensity: Number of cigarettes smoked per day (current + former smokers) | All participants | 53 | 58.9 ± 6.63 | 0.44, 95% CI 0.34 to 0.54 | <0.001 | '70.1 | 1.08 % |
|  | Women | 53 | 58.9 ± 6.64 | 0.52, 95% CI 0.40 to 0.65 | <0.001 | '70.4 | 1.60 % |
|  | Men | 53 | 58.9 ± 6.62 | 0.26, 95% CI 0.071 to 0.44 | 0.007 | '7.33 | 0.31 % |

^a^  Number of SNPs (among those identified in the GWAS by Liu M et al.) that were accessible in our database.

^b^ Unstandardized genetic risk score (sum of effect alleles across included SNPs).

# **Supplementary Table S7.** Two-sample Mendelian randomization analyses: main analysis (inverse variance weighted method) and sensitivity analyses (MR-Egger, weighted median, weighted mode) to validate the lack of horizontal pleiotropy

|  | SNPs | Inverse variance weighted | | MR-Egger | | Weighted median | | Weighted mode | | MR-Egger  intercept  (*p*-value) | Cochran’s Q  (*p*-value) | Rücker’s Q’  (*p*-value) |
| --- | --- | --- | --- | --- | --- | --- | --- | --- | --- | --- | --- | --- |
|  |  | OR [95% CI] | *p*-value | OR [95% CI] | *p*-value | OR [95% CI] | *p*-value | OR [95% CI] | *p*-value |  |  |  |
| Smoking initiation | 353 | 1.10  (1.06 to 1.13) | <0.001 | 1.09  (0.95 to 1.25) | 0.223 | 1.10  (1.05 to 1.15) | <0.001 | 1.06  (0.93 to 1.21) | 0.412 | 0.954 | 486  (*p*<0.001) | 486  (*p*<0.001) |
| Smoking intensity | 52 | 1.01  (0.95 to 1.09) | 0.671 | 1.03  (0.91 to 1.16) | 0.696 | 1.00  (0.91 to 1.09) | 0.920 | 1.02  (0.92 to 1.12) | 0.756 | 0.851 | 81.1  (*p*=0.005) | 81.1  (*p*=0.004) |
| Smoking cessation | 24 | 0.96  (0.90 to 1.02) | 0.197 | 0.82  (0.69 to 0.97) | 0.030 | 0.93  (0.85 to 1.03) | 0.161 | 0.87  (0.75 to 1.01) | 0.073 | 0.055 | 19.3  (*p*=0.682) | 15.2  (*p*=0.852) |

# **Supplementary Table S8**. Associations of GRSs of smoking-related traits with odds of COVID-19 infection in never smokers (non-relevance sensitivity analyses)

|  |  | Odds of COVID-19 infection  (OR per +1 SD in GRS) | *p*-value |
| --- | --- | --- | --- |
| GRS of smoking intensity | All participants | 0.99, 95% CI 0.94 to 1.05 | 0.773 |
|  | Women | 1.00, 95% CI 0.93 to 1.07 | 0.990 |
|  | Men | 0.98, 95% CI 0.89 to 1.07 | 0.616 |
| GRS of smoking cessation | All participants | 1.06, 95% CI 0.99 to 1.13 | 0.102 |
|  | Women | 1.03, 95% CI 0.95 to 1.12 | 0.501 |
|  | Men | 1.13, 95% CI 1.00 to 1.29 | 0.050 |

# **Supplementary Table S9**. Associations between genetic risk scores for smoking-related traits and COVID-19 infection risk factors with information available in MoBa

|  | Association with years of education  (β per +1 point in GRS, 95% CI) | *p*-value | Association with body mass index  (β per +1 point in GRS, 95% CI) | *p*-value |
| --- | --- | --- | --- | --- |
| GRS for smoking initiation | -0.016 (-0.018 to -0.014) | <0.001 | 0.015 (0.012 to 0.018) | <0.001 |
| GRS for smoking intensity | -0.006 (-0.009 to -0.002) | 0.001 | 0.008 (0.003 to 0.012) | 0.002 |
| GRS for smoking cessation | -0.016 (-0.026 to -0.005) | 0.003 | 0.004 (-0.010 to 0.019) | 0.562 |
